# Metabolic nuclear receptors coordinate energy metabolism to regulate Sox9^+^ hepatocyte fate

**DOI:** 10.1016/j.isci.2021.103003

**Published:** 2021-08-19

**Authors:** Shenghui Liu, Dan Qin, Yi Yan, Jiayan Wu, Lihua Meng, Wendong Huang, Liqiang Wang, Xiangmei Chen, Lisheng Zhang

**Affiliations:** 1 College of Veterinary Medicine/Bio-medical Center, Huazhong Agricultural University, Wuhan, Hu Bei 430070, China; 2 Department of Diabetes Complications and Metabolism, Diabetes and Metabolism Research Institute, Beckman Research Institute, City of Hope National Medical Center, Duarte, CA 91010, USA; 3 Department of Nephrology, Chinese PLA General Hospital, Chinese PLA Institute of Nephrology, State Key Laboratory of Kidney Diseases, National Clinical Research Center for Kidney Diseases, 28th Fuxing Road, Beijing 100853, China

**Keywords:** Human metabolism, Cellular physiology, Molecular physiology, Cell biology

## Abstract

Recent research has indicated the adult liver Sox9^+^ cells located in the portal triads contribute to the physiological maintenance of liver mass and injury repair. However, the physiology and pathology regulation mechanisms of adult liver Sox9^+^ cells remain unknown. Here, PPARα and FXR bound to the shared site in Sox9 promoter with opposite transcriptional outputs. PPARα activation enhanced the fatty acid β-oxidation, oxidative phosphorylation (OXPHOS), and adenosine triphosphate (ATP) production, thus promoting proliferation and differentiation of Sox9^+^ hepatocytes along periportal (PP)-perivenous (PV) axis. However, FXR activation increased glycolysis but decreased OXPHOS and ATP production, therefore preventing proliferation of Sox9^+^ hepatocytes along PP-PV axis by promoting Sox9^+^ hepatocyte self-renewal. Our research indicates that metabolic nuclear receptors play critical roles in liver progenitor Sox9^+^ hepatocyte homeostasis to initiate or terminate liver injury-induced cell proliferation and differentiation, suggesting that PPARα and FXR are potential therapeutic targets for modulating liver regeneration.

## Highlights

• PPARα promotes Sox9 expression and FXR inhibits Sox9 expression
• PPARα promotes proliferation and differentiation of Sox9^+^ hepatocytes
• FXR promotes Sox9^+^ hepatocyte self-renewal
• PPARα and FXR coordinate energy metabolism to regulate Sox9^+^ hepatocyte fate

## Graphical abstract

**Figure:**
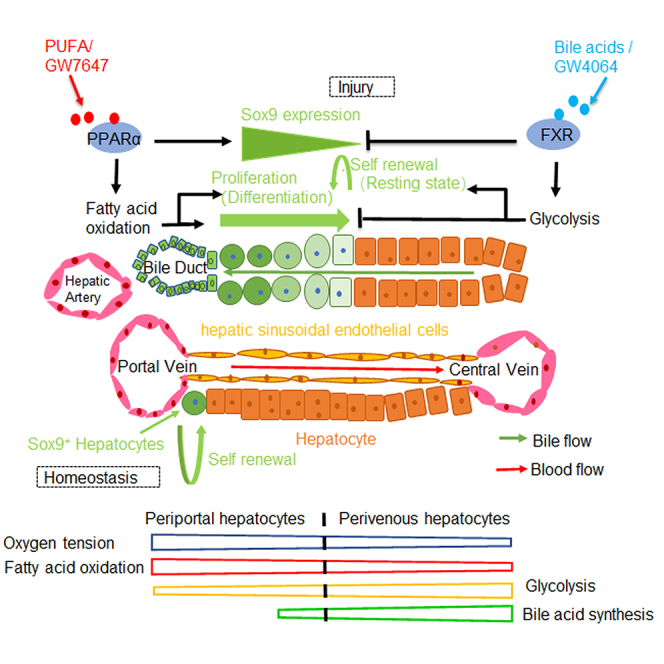

## Introduction

The liver is unique in its ability to regenerate in response to injury. A number of evolutionary safeguards allow the liver to continue to perform its complex functions despite severe injury (Mao et al., 2014). Recent studies have revealed that during liver injury, new hepatocytes arise by replication of hybrid hepatocytes (HybHP). The HybHPs expressed both hepatocyte nuclear factor 4 alpha (Hnf4α) and low levels of Sox9 which was expressed in adjacent biliary ductules. These HybHPs undergo extensive proliferation to replenish the liver mass after chronic hepatocyte-depleting injury (Font-Burgada et al., 2015).

The heterogeneity and demarcated metabolic zones of the liver enable hepatocytes flexible adaption to different circumstances (Wu et al., 2020). Nutrient- and oxygen-rich blood flows from periportal (PP) zone to pericentral zone, which causes hepatocytes to be exposed to different concentration of oxygens, hormones, and nutrients along the portocentral axis (Jiao et al., 1999). PP hepatocytes are responsible for gluconeogenesis and fatty acid β-oxidation (FAO), in contrast, pericentral hepatocytes are more involved in glycolysis and lipogenesis (Hijmans et al., 2014).

Metabolic nuclear receptors are key integrators of metabolic responses (Francis et al., 2003). PPARα and FXR have been reported to play the opposite roles in nutrient responses (Lee et al., 2014). For example, PPARα and FXR exhibited opposite effects on gluconeogenesis and lipid metabolism (Preidis et al., 2017), suggesting both broad functional interactions and additional counteracting metabolic effects.

Previous research has also shown that PPARα plays an important role in hepatocyte proliferation (Xie et al., 2019). Carnitine palmitoyl transferase 1a (Cpt1a) is a target gene of PPARα and a rate-limiting enzyme of FAO in PP hepatocytes (Berndt et al., 2021; Nakamura et al., 2014). In addition, PPARα is a nuclear hormone receptor that promotes FAO and oxidative phosphorylation (OXPHOS) (Pawlak et al., 2015). FAO is functionally and physically linked to OXPHOS: the reducing equivalents produced by FAO are directly used by the electron transport chain to fuel oxidative phosphorylation (Khoa et al., 2020). Furthermore, it is reported that mitochondria supports cellular proliferation by producing adenosine triphosphate (ATP) in OXPHOS to meet the bioenergetic demands of the cell (Yao et al., 2019).

FXR activation promotes post-injury liver repair and alleviates liver regeneration defect (Zhang et al., 2012b). Furthermore, FXR exerts direct effects on metabolic pathways, including suppression of both gluconeogenesis and lipid metabolism (Preidis et al., 2017). It is reported that FXR induces pyruvate dehydrogenate kinase 4(PDK4) expression in rat and human hepatocytes (Preidis et al., 2017), indicating that FXR may also promote glycolysis. Down-regulation of PDK4 is critical for the metabolic shift from glycolysis to OXPHOS during syncytialization (Stacpoole, 2017).

Coordinated regulation of metabolism and cell division during tissue renewal and regeneration is a prerequisite for tissue recovery after injury (Locasale and Cantley, 2011). In the normal liver regeneration through compensatory cellular hyperplasia, mitochondrial OXPHOS is necessary for hepatocyte proliferation (Caldez et al., 2018). Recent studies have shown that glycolysis contributes to termination of liver regeneration (Wang et al., 2020). In addition, glycolysis plays a critical role in the maintenance and induction of pluripotent stem cells (Cao et al., 2015). Studies have demonstrated that metabolic reprogramming is the driving force of cell transition (Takahashi et al., 2007; Takahashi and Yamanaka, 2006), and metabolic pathways influence stem cell function and fate (Ryall et al., 2015). Above all, we thus hypothesized that nutrient sensing metabolic nuclear receptors may maintain hepatic homeostasis by controlling a metabolic reprogramming.

In this study, we found that PPARα and FXR regulated Sox9 expression by binding to shared site in Sox9 promoter with opposite transcriptional outputs. PPARα activation promoted proliferation and differentiation of Sox9^+^ hepatocytes. FXR activation prevented the proliferation of Sox9^+^ hepatocytes by promoting self-renewal of Sox9^+^ hepatocytes. Our results provide an insight into the physiological functions of PPARα and FXR and the potential therapeutic roles of the metabolic nuclear receptors through manipulation of liver cell fate.

## Results

### PPARα activation induces the transcription of Sox9 and FXR activation suppresses the transcription of Sox9 *in vitro*

**Figure 1:**
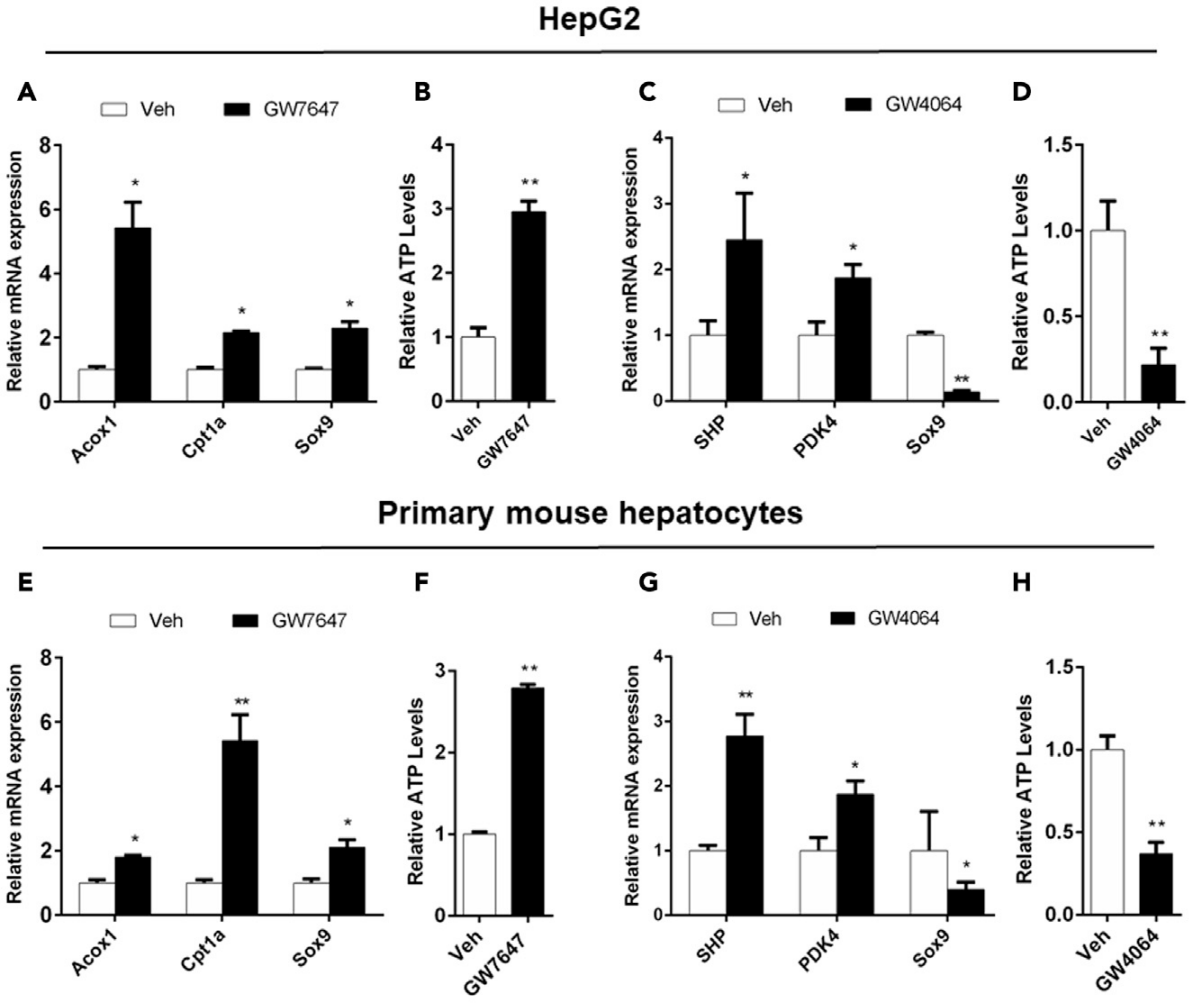
PPARα activation increases Sox9 expression and ATP abundance, and FXR suppresses Sox9 expression and ATP abundance *in vitro* (A) QRT-PCR analysis of Acox1, Cpt1a, and Sox9 expression in HepG2 cells. (B) ATP concentration measurements on HepG2 cells. (C) QRT-PCR analysis of SHP, PDK4, and Sox9 expression in HepG2. (D) ATP concentration measurements on HepG2. (E) QRT-PCR analysis of Acox1, Cpt1a, and Sox9 expression in primary mouse hepatocytes. (F) ATP concentration measurements on primary mouse hepatocytes. (G) QRT-PCR analysis of SHP, PDK4, and Sox9 expression in primary mouse hepatocytes. (H) ATP concentration measurements on primary mouse hepatocytes. Data are expressed as means ± SD. Comparisons between two groups were performed using the two-tailed Student's t-test. Significant difference is presented at the levels of ∗p < 0.05 and ∗∗p < 0.01.

PPARα, FXR and Sox9 were expressed in HepG2 cells (Ghonem et al., 2014; Ramos Pittol et al., 2020). To determine the regulation of Sox9 expression by PPARα, HepG2 cells were treated with PPARα agonist GW7647, followed by quantitative real-time PCR analysis of gene expression. Acyl-CoA Oxidase 1(Acox1) and Cpt1a were target genes of PPARα. The significant up-regulation of Acox1 and Cpt1a expression demonstrated the activation of PPARα (Figure 1A). After PPARα activation, Sox9 expression was up-regulated (Figure 1A). Western blot analysis supported this result (Figure S1A). Significant increase in ATP levels was observed after pharmacological activation of PPARα (Figure 1B). These results showed that PPARα activation increased the expression of Sox9 and FAO, thus enhancing OXPHOS and ATP levels. In addition, HepG2 cells were treated with FXR agonist GW4064, followed by quantitative real-time PCR analysis of gene expression. Small heterodimeric partner (SHP) and PDK4 were FXR target gene (Zhang et al., 2009). The significant up-regulation of SHP and PDK4 expression demonstrated the activation of FXR (Figure 1C). After FXR activation, Sox9 expression levels were decreased (Figure 1C). Western blot analysis also further confirmed this result (Figure S1A). FXR activation was also found to decrease ATP levels (Figure 1D). These results indicated that FXR activation suppressed the expression of Sox9, followed by the increase in glycolysis and the decrease in OXPHOS and ATP levels.

Primary mouse hepatocytes were also treated with PPARα agonist GW7647 or FXR agonist GW4064, and the results further indicated that PPARα or FXR regulated Sox9 transcription (Figures 1E–1H and S1B).

### PPARα increases the expression of Sox9 and FXR suppresses the expression of Sox9 *in vivo*

**Figure 2:**
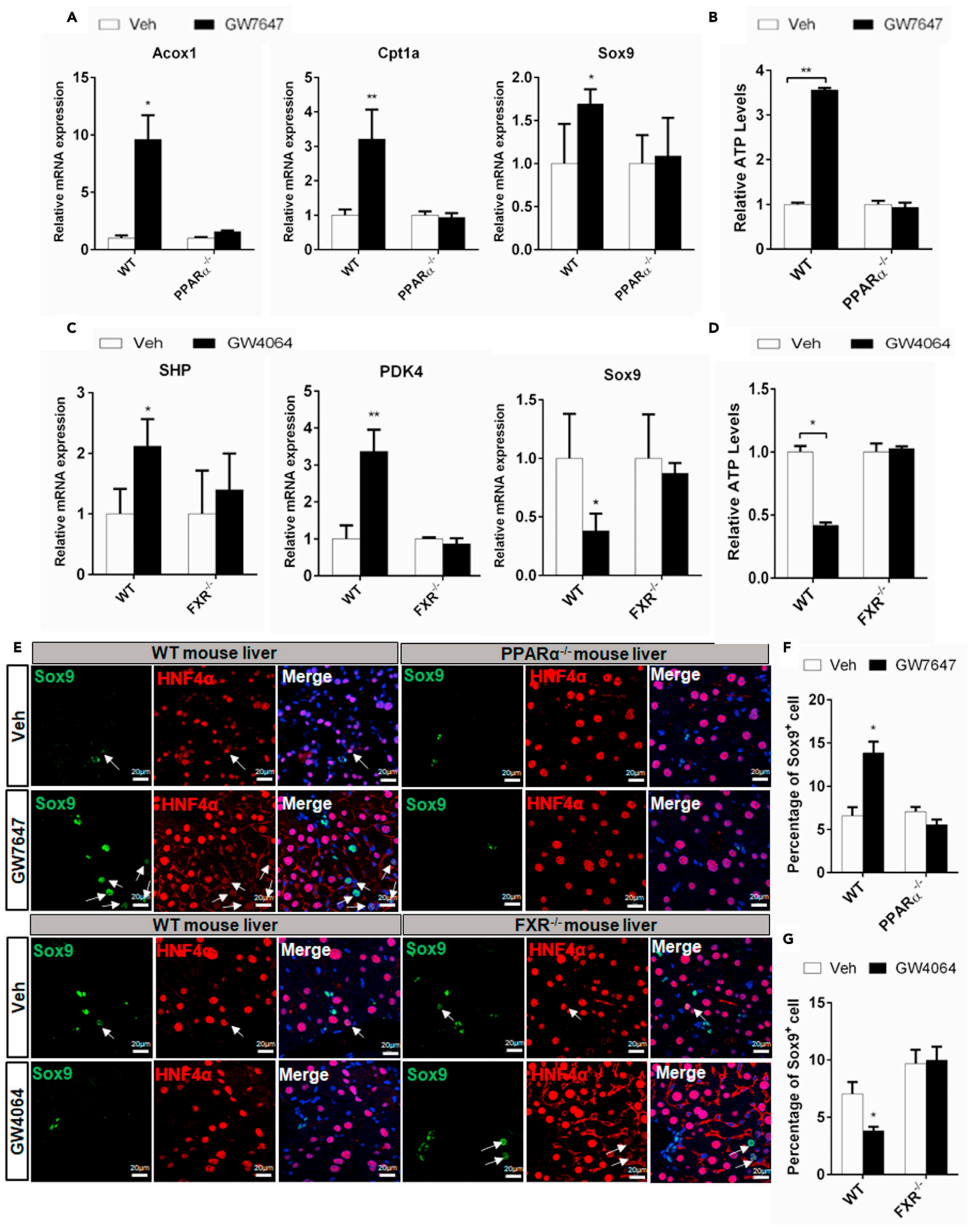
PPARα and FXR oppositely regulate Sox9 expression and ATP levels in vivo WT, PPARα^−/−^ and FXR^−/−^ mice were orally treated with Veh, GW7647, or GW4064 twice a day for two days. (A) Hepatic expression levels of Acox1, Cpt1a, and Sox9 were determined by QRT-PCR analysis (n = 5). (B) ATP concentration measurements of liver samples (n = 5). (C) Hepatic expression levels of SHP, PDK4, and Sox9 were determined by QRT-PCR analysis (n = 5). (D) ATP concentration measurements of liver samples (n = 5). (E) Sox9/Hnf4α double staining was performed. Arrows depict Sox9^+^Hnf4α^+^ cells. Scale bar represents 20μm. (F) Graphs show percentages of Sox9^+^ cells (n = 5). (G) Graphs show percentages of Sox9^+^ cells (n = 5). Data are expressed as means ± SD. Comparisons between two groups were performed using the two-tailed Student's t-test. Significant difference is presented at the levels of ∗p < 0.05 and ∗∗p < 0.01.

To decipher investigate whether PPARα and FXR specifically regulated Sox9 transcription *in vivo*. WT (wild-type), PPARα^−/−^, and FXR^−/−^ mice were treated with the PPARα agonists GW7467 or FXR agonist GW4064, respectively. The results showed that PPARα activation significantly increased the expression of Sox9 in WT mice but not in PPARα^−/−^ mice (Figure 2A), which was in line with Western blot analysis results (Figure S1C). Also, Cpt1a expression and ATP production were induced in WT mice, but no change was observed in PPARα^−/−^ mice (Figures 2A and 2B). The FXR activation suppressed the expression of Sox9 in WT mice, but no change was observed in FXR^−/−^ mice (Figure 2C), which was consistent with Western blot analysis results (Figure S1D). In addition, FXR activation was found to induce PDK4 expression and decrease ATP levels in WT mice, but no change was detected in FXR^−/−^ mice (Figures 2C and 2D). These results further indicated that PPARα and FXR regulated Sox9 transcription.

Immunofluorescence (IF) analysis illustrated a small number of Sox9^+^/Hnf4α^+^ hepatocytes around PP area in WT, PPARα^−/−^, and FXR^−/−^ mice. Furthermore, after GW7647 treatment, the proliferation of Sox9^+^/Hnf4α^+^ hepatocytes were observed in WT mice, but not in PPARα^−/−^ mice. But, after GW4064 treatment, only few Sox9^+^/Hnf4α^+^ hepatocytes in WT, and no change was observed in FXR^−/−^ mice (Figure 2E). The graphs showed that PPARα activation increased the percentages of Sox9^+^ cell in WT mice but not in PPARα^−/−^ mice (Figure 2F), whereas FXR activation decreased the percentages of Sox9^+^ cells in WT mice but not in FXR^−/−^ mice (Figure 2G). These results suggested that PPARα and FXR coordinately maintained Sox9^+^ hepatocytes balance under physiological conditions.

### Sox9 is a target of PPARα and FXR

**Figure 3:**
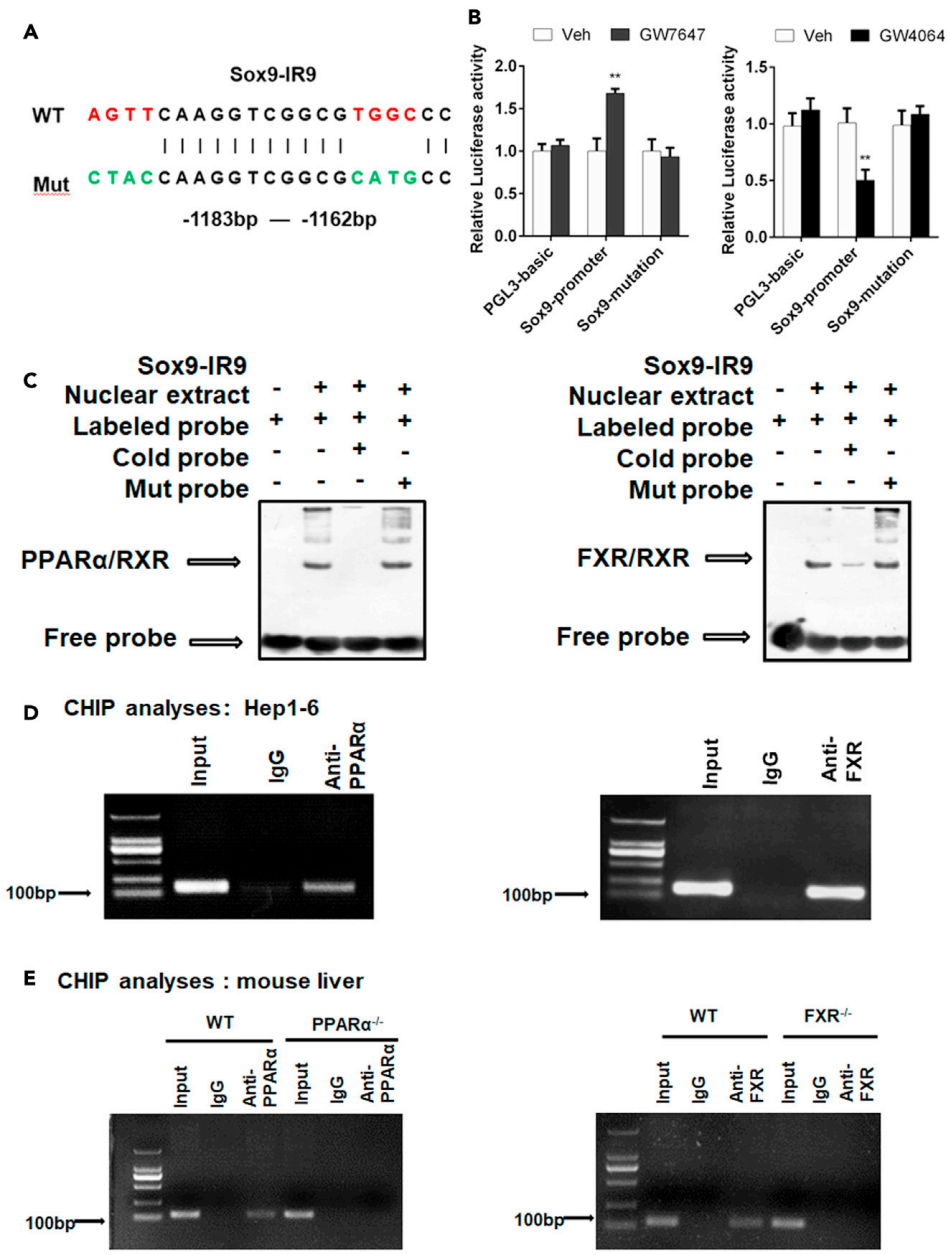
Sox9 is a target of PPARα and FXR (A) Potential PPRE and FXRE IR9 in the Sox9 promoter region were predicted using online algorithm (http://www.nubiscan.unibas.ch/). (B) Functional role of IR9 motif in the regulatory region of mouse Sox9 for PPARα and FXR activity in HepG2. (C) EMSA assays were performed on mouse liver tissue. (D) ChIP experiments were performed on Hep1-6 cells. (E) ChIP experiments were performed on mouse liver tissue. Data are expressed as means ± SD. Comparisons between two groups were performed using the two-tailed Student's t-test. Significant difference is presented at the levels of ∗p < 0.05 and ∗∗p < 0.01.

As can be seen from above results, direct transcriptional effects are the most possible explanation for the impact of both PPARα and FXR on Sox9. We then analyzed Sox9 at potential PPARα and FXR binding sites using an online algorithm (NUBISCAN, http://www.nubiscan.unibas.ch/) (Figure 3A). Based on the prediction, the Sox9 promoter fragments were amplified by PCR using mouse genomic DNA as a template. Then, we performed site-directed mutation of the elements. Luciferase reporter assays indicated that GW7647 treatment increased reporter activity, and that GW4064 treatment decreased reporter activity in HepG2 and Hep1-6 cells (Figures 3B and S2). After promoter mutation, reporter activity remained unchanged even if PPARα or FXR was activated (Figures 3B and S2). To determine whether specific elements of Sox9 promoter can function as a PPARα response element (PPRE) or FXR response element (FXRE), EMSA was performed in mouse liver. WT mice were treated with vehicle, GW7647, or GW4064. EMSA revealed that the interaction of labeled probe with the nuclear extracts of mouse liver that was treated with GW7647 or GW4064 yielded a DNA/protein shift band of the expected mobility. This binding was specific because it was competitively inhibited by the addition of excess unlabeled (cold) probes, rather than by mut probes (Figure 3C). To further confirm the interaction of PPARα or FXR with the IR9 element in the SOX9 promoter, we performed ChIP assays. As shown in Figure 3D, the anti-PPARα antibody precipitated the DNA fragment containing the IR9 element and the anti-FXR antibody precipitated the DNA fragment containing the IR9 element in Hep1-6 cells. In addition, The ChIP results indicated that both anti-PPARα antibody and anti-FXR antibody precipitated the same DNA fragment containing the IR9 element in WT mouse liver, whereas no precipitation of this DNA fragment was observed in PPARα^−/−^, and FXR^−/−^ mouse liver (Figure 3E). Overall, both *in vitro* and *in vivo* experiment results demonstrated that PPARα or FXR bound to the same Sox9 promoter element, but these two metabolic nuclear receptors mediated opposite transcriptional outputs.

### PPARα induces Sox9 expression and FAO, and FXR suppresses Sox9 expression and increases the glycolysis after CCl_4_-induced chronic mouse liver injury

**Figure 4:**
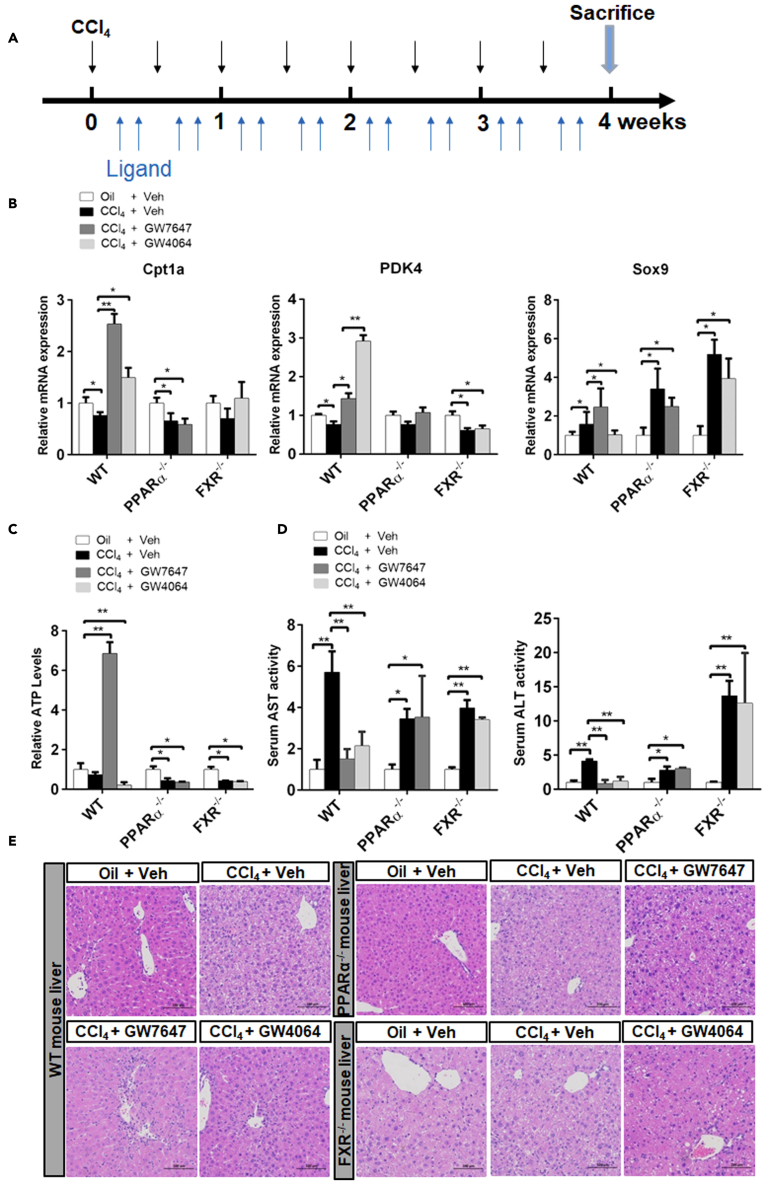
PPARα activation induces Sox9 expression and FAO, and FXR suppresses Sox9 expression and increases the glycolysis after CCl_4_-induced chronic liver injury (A) The 6- to 8-week-old WT mice, PPARα^−/−^ mice, or FXR^−/−^ mice were received intraperitoneal paraffin oil injection (control group) or CCl_4_ injection twice per week for four weeks and these mice were orally gavaged with either Veh, GW7647, or GW4064 four times a week for four weeks. BrdU was injected twice per day for two days before sacrifice. (B) Hepatic expression levels of Cpt1a, PDK4, and Sox9 were determined by QRT-PCR analysis (n = 5). (C) ATP concentration measurements of liver samples (n = 5). (D) Serum AST and ALT levels were measured (n = 5). (E) Histopathological analysis of representative mouse liver samples following H&E staining. Scale bar represents 100μm. Data are expressed as means ± SD. Comparisons between multiple groups were performed using ordinary one-way ANOVA with the Dunnett's multiple comparison test. Significant difference is presented at the levels of ∗p < 0.05 and ∗∗p < 0.01.

As mentioned above, PPARα and FXR functioned coordinatively to maintain Sox9^+^ hepatocyte balance under physiological conditions. Next, we investigated whether PPARα and FXR played similar roles in CCl_4_-induced liver injury repair. WT, PPARα^−/−^, or FXR^−/−^ mice received intraperitoneal paraffin oil injections (control group) or CCl_4_ injections twice a week for four weeks. And were orally gavaged with either vehicle, GW7647, or GW4064 four times per week for four weeks. BrdU was injected twice a day for two days before sacrifice (Figure 4A). Compared to control group, CCl_4_ treatment group exhibited higher levels of Sox9 expression. After CCl_4_-induced injury, GW7647 treatment induced the expression of Sox9 in WT but no change in PPARα^−/−^ mice. On the contrary, GW4064 treatment significantly decreased expression level of Sox9 in CCl_4_-induced injury in WT mice but no change in FXR^−/−^ mice (Figure 4B). CCl_4_ treatment group exhibited lower levels of PDK4 and Cpt1a expression and lower levels of ATP (Figures 4B and 4C). PPARα activation was found to induce Cpt1a expression and increase ATP levels in WT but no change in PPARα^−/−^ mice (Figures 4B and 4C). FXR activation was found to induce PDK4 expression and decrease ATP levels in WT, but no change in PDK4 or ATP was found in FXR^−/−^ mice (Figures 4B and 4C). CCl_4_ treatment induced hepatotoxicity, and the level of hepatic transaminase in CCl_4_ treatment group was observed to be significantly higher than that in control group (Figure 4D). First injury by CCl_4_ and subsequent treatment with GW7647 or GW4064 lowered significantly the levels of serum aspartate aminotransferase (AST) and alanine aminotransferase (ALT) in WT but not in PPARα^−/−^ and FXR^−/−^ mice (Figure 4D). In addition, first injury by CCl_4_ and subsequent treatment with GW7647 or GW4064 led to significantly improved tissue structure in WT but not in PPARα^−/−^ and FXR^−/−^ mice (Figure 4E). These results indicating that PPARα and FXR activation alleviated liver injury caused by CCl_4_. RNAscope® assays confirmed that CCl_4_-injured mice exhibited higher expression level of Sox9 in the PP area. The treatment with GW7647 further elevated Sox9 expression in CCl_4_-injured WT but not in PPARα^−/−^ mice, whereas GW4064 suppressed Sox9 expression in CCl_4_-injured WT but not in FXR^−/−^ mice (Figure S3). Integrated Optical Density analysis also confirmed the above result (Figure S3). These results indicated that PPARα activation increases Sox9 expression and FAO, while FXR activation suppresses Sox9 expression and increases the glycolysis to keep the proper level of the Sox9 expression in the injury repair.

### PPARα and FXR oppositely regulate proliferation of Sox9^+^ hepatocytes after CCl_4_-induced chronic liver injury

**Figure 5:**
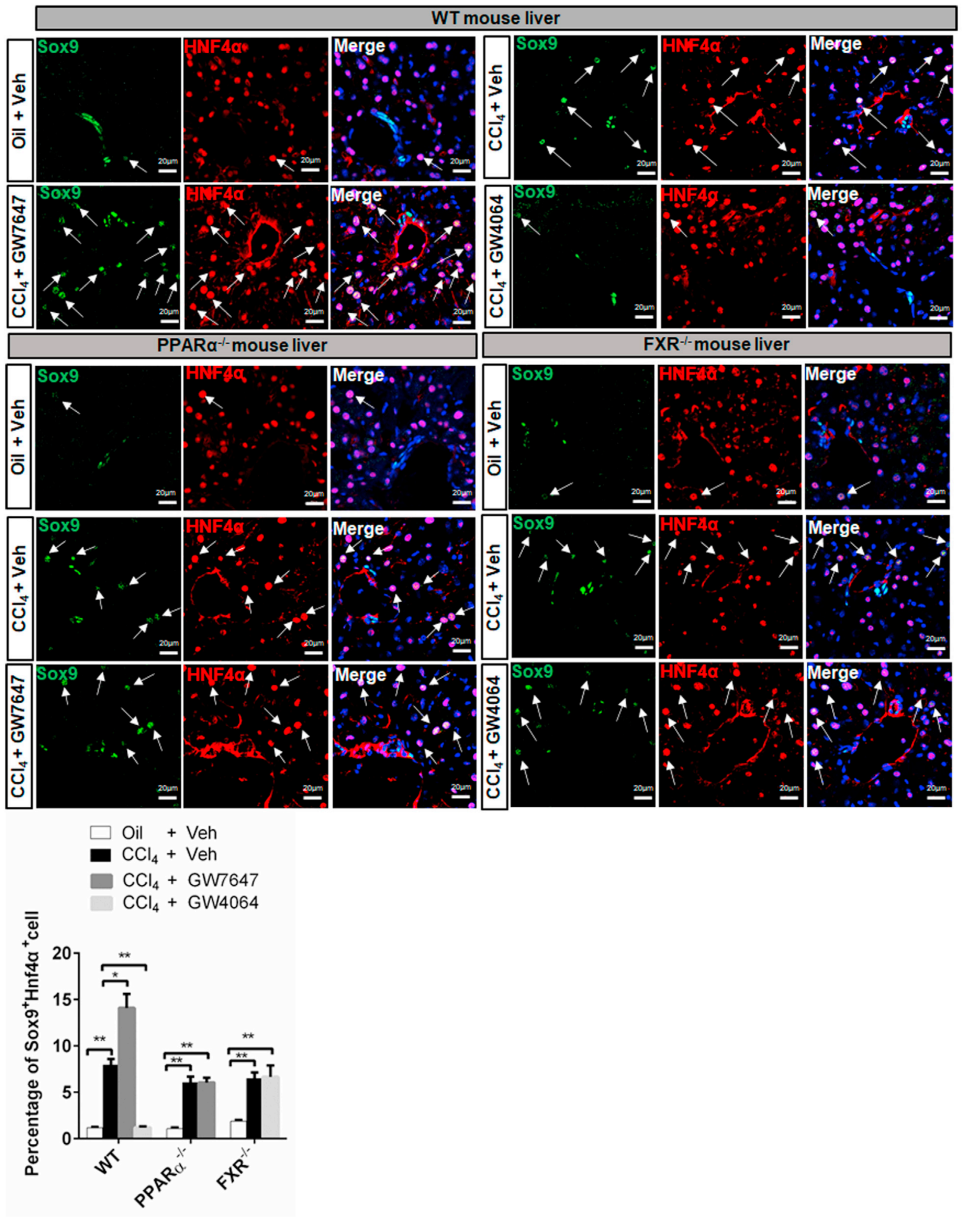
PPARα promotes, but FXR prevents proliferation of Sox9^+^ hepatocytes in CCl_4_-induced chronic liver injury model. The model of CCl_4_-induced chronic liver injury was described in Figure 4A. Sox9/Hnf4α double staining was performed. Arrows depict Sox9^+^Hnf4α^+^ cells. Graphs show percentages of Sox9^+^Hnf4α^+^ cell (n = 5). Scale bar represents 20μm. Data are expressed as means ± SD. Comparisons between multiple groups were performed using ordinary one-way ANOVA with the Dunnett's multiple comparison test. Significant difference is presented at the levels of ∗p < 0.05 and ∗∗p < 0.01.

IF analysis indicated that the number of Sox9^+^/Hnf4α^+^ hepatocytes around the PP area in CCl_4_ administration group was larger around the PP area than that in the control group. After GW7647 treatment, CCl_4_-injured mice exhibited a larger number of Sox9^+^/Hnf4α^+^ hepatocytes in WT mice but not in PPARα^−/−^ mice. After GW4064 treatment, CCl_4_-injured mice presented a smaller number of Sox9^+^/Hnf4α^+^ hepatocytes in WT mice, but not in FXR^−/−^ mice (Figure 5). The number of Sox9^+^/BrdU^+^ cells in CCl_4_ -injured group was larger than the control group. CCl_4_-injured GW7647-treated mice exhibited much more Sox9^+^/BrdU^+^ cells than the merely CCl_4_-injured mice, but this result was not observed in PPARα^−/−^ mice. FXR activation led to the decreased number of Sox9^+^/BrdU^+^ cell in CCl_4_-injured WT, but no change in Sox9^+^/BrdU^+^ cell number was observed in FXR^−/−^ mice (Figure S4). CCl_4_-injured group exhibited few CK19^+^/BrdU^+^ cell in WT, PPARα^−/−^ or FXR^−/−^ mice. After GW7647 or GW4064 treatment, almost no change of CK19^+^/BrdU^+^ cell number was observed in CCl_4_-injured WT, PPARα^−/−^ or FXR^−/−^ mice (Figure S5). These results further indicated that PPARα activation induced the expression of Sox9, thus promoting the proliferation of Sox9^+^ hepatocytes, and that FXR activation suppressed the expression of Sox9, further preventing the proliferation of Sox9^+^ hepatocytes.

### PPARα promotes and FXR prevents the proliferation of Sox9^+^ hepatocytes after CCl_4_-induced chronic liver injury in Sox9-Cre^ERT2^; Rosa26-mTmG mice

**Figure 6:**
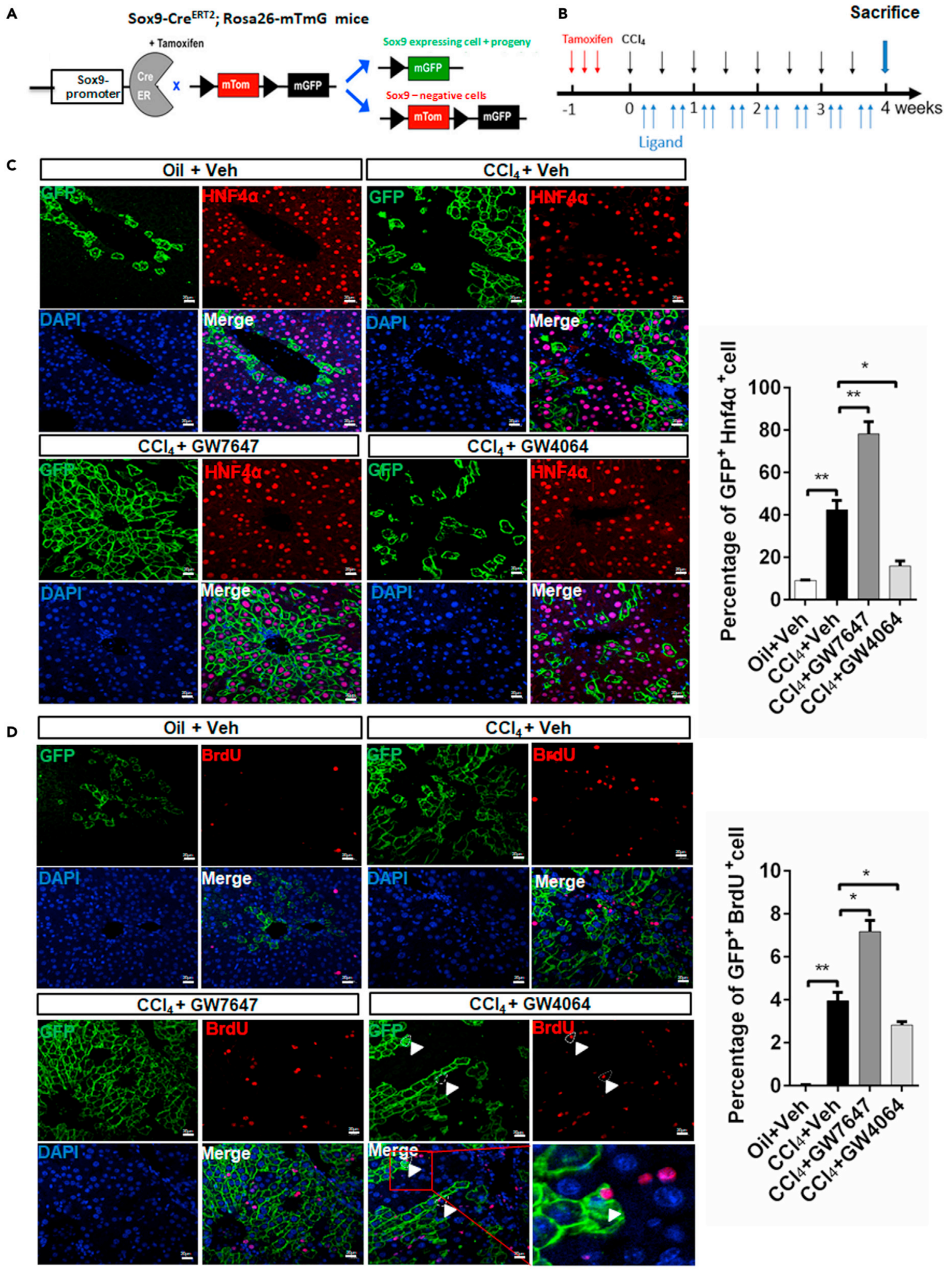
PPARα activation promotes proliferation of Sox9^+^ hepatocytes, and FXR suppresses proliferation of Sox9^+^ hepatocytes after CCl_4_-induced chronic liver injury in Sox9-Cre^ERT2^; Rosa26-mTmG mice (A) Schematic diagram showing mTom/mGFP reporter gene expression in the absence and presence of tamoxifen (TAM)-inducible Cre-mediated recombination. (B) Sox9-Cre^ERT2^; Rosa26-mTmG mice were intraperitoneally injected with a single dose of tamoxifen once per day for three days before treatment. The Sox9-Cre^ERT2^; Rosa26-mTmG mice were received intraperitoneal paraffin oil injection (control group) or CCl_4_ injection twice per week for four weeks and these mice were orally gavaged with either Veh, GW7647, or GW4064 four times a week for four weeks. BrdU was injected twice per day for two days before sacrifice. (C) GFP(Sox9)/Hnf4α double staining was performed. Graphs show percentages of GFP^+^Hnf4α^+^ cell (n = 5). Scale bar represents 20μm. (D) GFP(Sox9)/BrdU double staining was performed. Arrowheads depict the asymmetric division. Graphs show percentages of GFP^+^BrdU^+^ cell (n = 5). Scale bar represents 20μm. Data are expressed as means ± SD. Comparisons between multiple groups were performed using ordinary one-way ANOVA with the Dunnett's multiple comparison test. Significant difference is presented at the levels of ∗p < 0.05 and ∗∗p < 0.01.

To further elaborate the influence of PPARα and FXR on Sox9^+^ hepatocytes, we used Sox9-Cre^ERT2^; Rosa26-mTmG mice in our experiments. Schematic diagram showing mTom/mGFP reporter gene expression in the absence and presence of tamoxifen-inducible Cre-mediated recombination (Figure 6A). Sox9-Cre^ERT2^; Rosa26-mTmG mice were injected with a single dose of tamoxifen (100mg/kg body weight) one time per day for three days before treatment. Subsequently, Sox9-Cre^ERT2^; Rosa26-mTmG mice were received paraffin oil injections (control group) or CCl_4_ injections twice per week for four weeks, and they were orally gavaged with either vehicle, GW7647, or GW4064 four times a week for four weeks. Before sacrifice, BrdU was injected twice on daily basis for two days (Figure 6B). After tamoxifen administration, Sox9^+^ cells and their progenies were labeled by green fluorescent protein (GFP). The lineage tracing experiments demonstrated that GFP^+^/Hnf4α^+^hepatocytes were located in PP area and that CCl_4_-induced chronic liver injury promoted the proliferation of GFP^+^/Hnf4α^+^hepatocytes. PPARα activation promoted the propagation of GFP^+^/Hnf4α^+^ hepatocyte descendants, whereas FXR activation inhibited the propagation of GFP^+^/Hnf4α^+^ hepatocyte descendants (Figure 6C). Compared merely CCl_4_-injured group, CCl_4_-injured GW7647-treated group exhibited a larger number of GFP^+^/BrdU^+^ hepatocytes. GW4064 treatment decreased the number of GFP^+^/BrdU^+^ hepatocytes but FXR activation promoted GFP^+^ hepatocyte asymmetric division (Figure 6D). These results indicated that PPARα activation promoted the proliferation of Sox9^+^ hepatocytes, whereas FXR activation prevented the proliferation of Sox9^+^ hepatocytes, suggesting that PPARα might be the driving force to promote the proliferation and differentiation of Sox9^+^ hepatocytes, while FXR inhibited this process and made Sox9^+^ hepatocytes return to quiescent state to terminate the Sox9^+^ hepatocyte-mediated liver regeneration.

### PPARα induces proliferation and differentiation of Sox9^+^ hepatocytes by enhancing FAO and OXPHOS and FXR promote self-renewal of Sox9^+^ hepatocytes by increasing glycolysis and inhibiting OXPHOS

**Figure 7:**
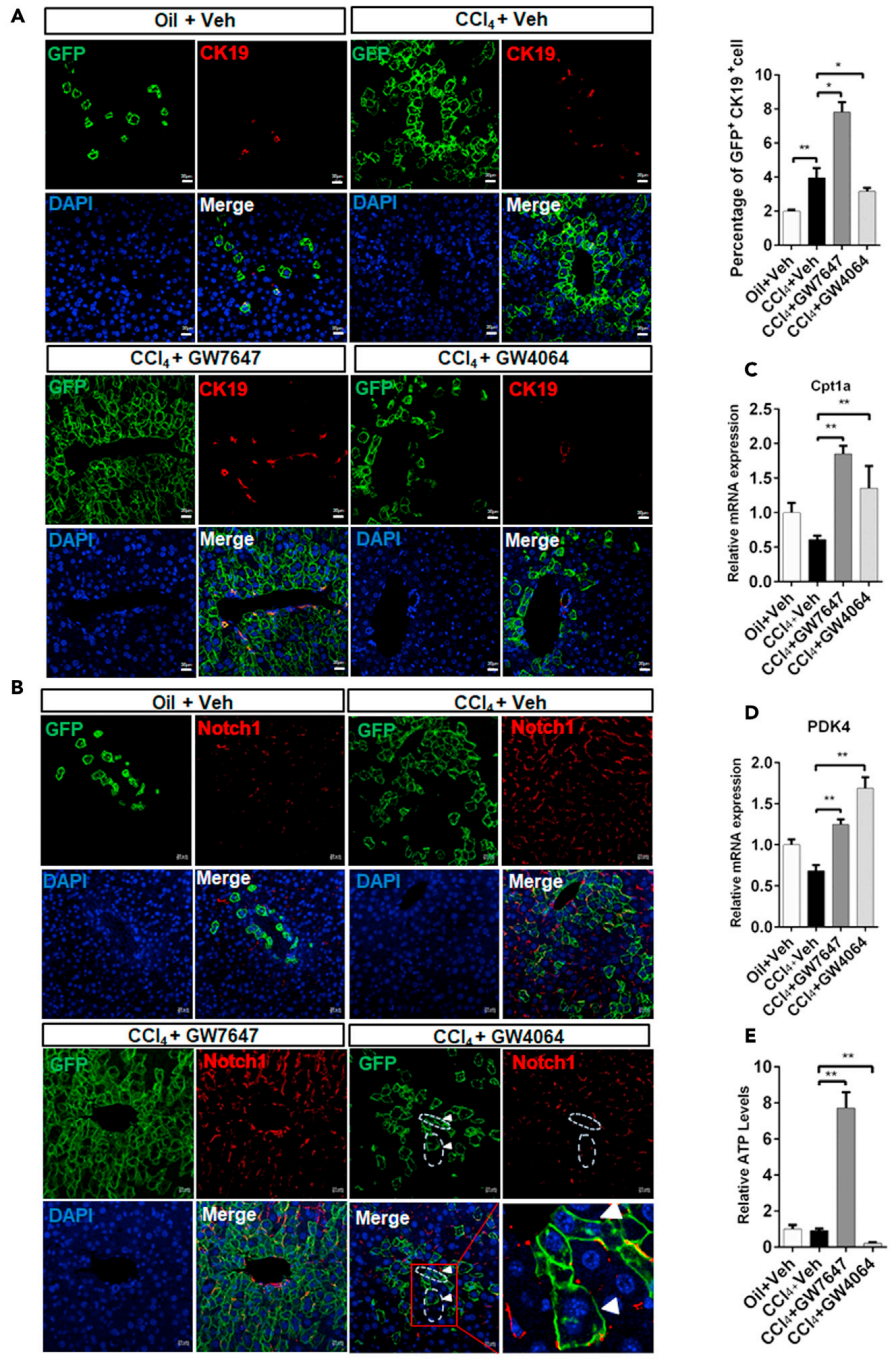
PPARα activation increases differentiation of Sox9^+^ hepatocytes, and FXR activation promotes self-renewal of Sox9^+^ hepatocytes in Sox9-Cre^ERT2^; Rosa26-mTmG mice The model of CCl_4_-induced chronic liver injury in Sox9-Cre^ERT2^; Rosa26-mTmG mice that was described in Figure 6B. (A) GFP(Sox9)/CK19 staining was performed in the indicated groups. Graphs show percentages of GFP^+^CK19^+^ cells (n = 5). Scale bar represents 20μm. (B) GFP(Sox9)/Notch1 staining was performed in the indicated groups. Scale bar represents 20μm. (C) Hepatic expression levels of Cpt1a were determined by QRT-PCR analysis (n = 5). (D) Hepatic expression levels of PDK4 were determined by QRT-PCR analysis (n = 5). (E) ATP concentration measurements of liver samples (n = 5). Data are expressed as means ± SD. Comparisons between multiple groups were performed using ordinary one-way ANOVA with the Dunnett's multiple comparison test. Significant difference is presented at the levels of ∗p < 0.05 and ∗∗p < 0.01.

**Figure 8:**
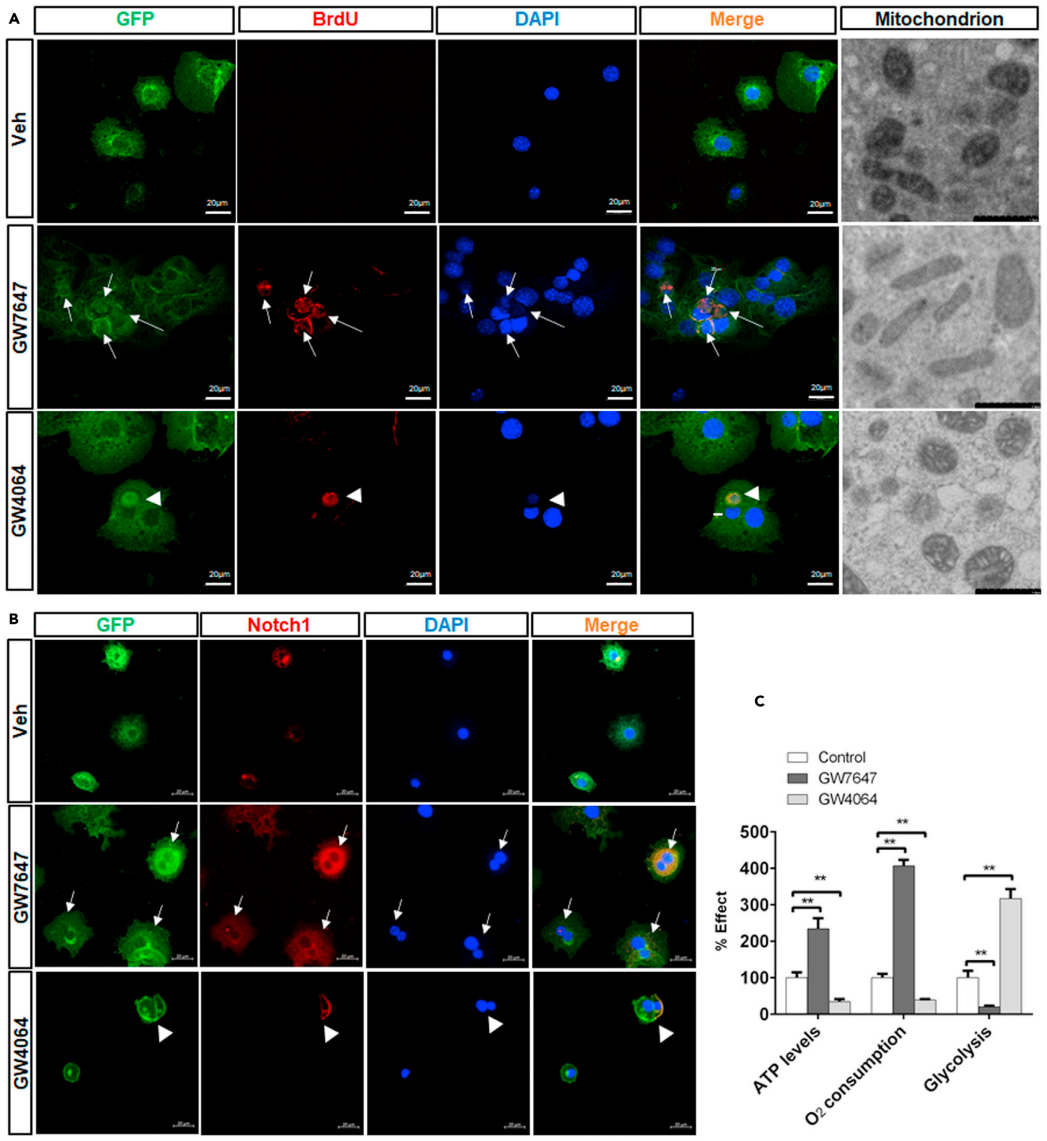
PPARα induces proliferation and differentiation of Sox9+ hepatocytes by enhancing OXPHOS, and FXR promote self-renewal of Sox9^+^ hepatocytes by increasing glycolysis and inhibiting OXPHOS (A) GFP^+^ primary mouse hepatocytes were stained with BrdU. Scale bar represents 20μm. The morphology of mitochondria in GFP^+^ hepatocytes. Scale bar represents 1μm. (B) GFP^+^ primary mouse hepatocytes were stained with Notch1. Scale bar represents 20μm. (C) ATP concentration, O_2_ consumption and Glycolysis measurements of primary mouse hepatocytes. Data are expressed as means ± SD. Comparisons between multiple groups were performed using ordinary one-way ANOVA with the Dunnett's multiple comparison test. Significant difference is presented at the levels of ∗p < 0.05 and ∗∗p < 0.01

Previous study showed that Sox9^+^ hepatocytes were differentiated into bile duct cells (Font-Burgada et al., 2015). Compared merely CCl_4_-injured group, CCl_4_-injured GW7647-treated group exhibited an increased number of GFP^+^/CK19^+^ hepatocytes but CCl_4_-injured GW4064-treated group displayed a decreased number of GFP^+^/CK19^+^ hepatocytes (Figure 7A). However, CCl_4_-injured group exhibited few CK19^+^/BrdU^+^ cell, and almost no change in CK19^+^/BrdU^+^ cell number was observed (Figure S6) after GW7647 or GW4064 treatment. These results suggest that PPARα activation promoted Sox9^+^ hepatocyte differentiation, whereas FXR activation inhibited Sox9^+^ hepatocyte differentiation. Our data have shown that FXR represses Notch1 expression and directs asymmetrical cell division of Sox9^+^ cells (Chen et al., 2021). Compared merely CCl_4_-injured group, Notch1 expression was decreased in CCl_4_-injured GW4064-treated group, and FXR activation enhanced GFP^+^ hepatocyte asymmetric division (Figure 7B). PPARα was found to induce Cpt1a expression and increased ATP levels (Figures 7C and 7E), and FXR was observed to induce PDK4 expression and decreased ATP levels (Figures 7D and 7E). GFP^+^ primary mouse hepatocytes from Sox9-Cre^ERT2^; Rosa26-mTmG mice were incubated with BrdU and treated with vehicle, GW7647, or GW4064. The results indicated that GW7647 treatment promoted proliferation of GFP^+^/BrdU^+^ hepatocytes, and that GW4064 treatment induced the asymmetric division of GFP^+^/BrdU^+^ hepatocytes (Figure 8A). The morphology of mitochondria in GFP^+^ primary mouse hepatocytes treated with Vehicle, GW7647 or GW4064 were detected. The results showed the morphology of the mitochondria in the control group was mainly globule-shaped. After GW7647 treatment, the morphology of mitochondria became elongated. After GW4064 treatment, the number of globule-shaped mitochondria increased (Figure 8A). GFP^+^ primary mouse hepatocytes from Sox9-Cre^ERT2^; Rosa26-mTmG mice were treated with Vehicle, GW7647, or GW4064 to carry out immunofluorescent analysis. The results showed that GW7647 treatment promoted proliferation of GFP^+^/Notch1^+^ hepatocytes, whereas GW4064 treatment induced the asymmetric division of GFP^+^/Notch1^+^ hepatocytes (Figure 8B). Next, effects of PPARα or FXR agonist on ATP, O_2_ consumption and glycolysis were measured in primary mouse hepatocytes. As shown in Figure 8C, GW7647 treatment increased ATP levels and O_2_ consumption, and decreased glycolysis, while, GW4064 decreased ATP levels and O_2_ consumption, but increased glycolysis.

These results indicated that PPARα promoted proliferation and differentiation of Sox9^+^ hepatocytes by increasing FAO and OXPHOS, and that FXR promoted self-renewal of Sox9^+^ hepatocytes by increasing glycolysis and inhibiting OXPHOS.

Taken together, PPARα and FXR oppositely controlled Sox9^+^ hepatocytes fate in homeostasis balance and injury repair via regulating FAO or glycolysis.

## Discussion

Under normal homeostatic conditions, hepatocyte renewal is a slow process (Carpentier et al., 2011). Sox9^+^ hepatocytes are able to exit the homeostasis and rapidly propagate and differentiate in response to tissue injury (Miyajima et al., 2014), but the related molecular signals and mechanisms have not been investigated. Our study indicated that PPARα activation increased Sox9^+^/Hnf4α^+^ cell numbers, whereas FXR activation decreased Sox9^+^/Hnf4α^+^ cell numbers. Moreover, PPARα and FXR bound to the shared sites in Sox9 promoters with opposite transcriptional outputs. Our results revealed complementary interlocking mechanisms by which PPARα and FXR regulated Sox9 expression to maintain liver homeostasis. Furthermore, in a mouse model of CCl_4_-induced chronic liver injury, PPARα activation increased the FAO, OXPHOS, and ATP levels, thus promoting proliferation and differentiation of Sox9^+^ hepatocytes along PP-perivenous (PV) axis; however, FXR activation enhanced the glycolysis, decreased OXPHOS and production of ATP, therefore preventing the proliferation of Sox9^+^ hepatocytes along PP-PV axis by promoting self-renewal or quiescent state of Sox9^+^ hepatocytes. Our findings highlighted the effect of PPARα and FXR on Sox9^+^ hepatocytes fate in hepatic homeostasis and injury repair.

Sox9 is an important nuclear transcription factor, which is closely related to cell proliferation and differentiation, and regulates the dynamic balance between stem cell homeostasis and differentiation (Antoniou et al., 2009; Font-Burgada et al., 2015; Furuyama et al., 2011). In addition, Sox9 is highly expressed in hepatocellular carcinoma (HCC) tissues, which promotes the self-renewal of HCC stem cells and affects the occurrence and differentiation of HCC (Larsimont et al., 2015). Under normal physiological conditions, it is indispensable in development of organs (Furuyama et al., 2011). Lipid was reported to affect cartilage development by regulating Sox9 (van Gastel et al., 2020). Metabolic switch was reported to have a significant effect on Sox9^+^ hair follicle stem cell (Kim et al., 2020). Our data have shown that Sox9^+^ hepatocytes properties are regulated by microRNAs during liver repair (Yan et al., 2020). Here, our results illustrated that metabolic nuclear receptor, PPARα and FXR, played critical roles in regulating Sox9^+^ hepatocyte dynamic fate during both physiological (maintenance) and pathological (repair) regeneration processes. It has to be mentioned that PPARα and FXR activation maybe also affect Sox9 expression in cholangiocytes, as well as in Sox9^+^ hepatocytes since the nuclear receptors are expressed in bile duct epithelial cells.

The liver occupies a strategic position for overall metabolic function in the body. Previous studies have shown that hepatocytes located in the PP and PV zones of the liver display a remarkable heterogeneity in the enzyme activity, metabolism functions, and gene expression (Braeuning et al., 2006; Kietzmann, 2017). PPARα is a key transcriptional regulator for FAO which is active exclusively in PP hepatocytes, Cpt1a, a well-known target gene of PPARα, is a rate-limiting enzyme of FAO in PP hepatocytes (Zhang et al., 2016), and FXR activation promotes the expressions of Cpt1a (Xi and Li, 2020). Bile acid synthesis from cholesterol takes place in PV hepatocytes, and cholesterol 7α-hydroxylase (Cyp7a1), as a key enzyme of this synthesis pathway, is preferentially expressed in PV hepatocytes (Braeuning et al., 2006; Li et al., 2013). It is well known that PPARα can be activated by polyunsaturated fatty acids (PUFAs) (Zhang et al., 2016), and FXR is activated by bile acids (Chiang and Ferrell, 2019). In addition, FAO preferentially occurs in the PP area (Kietzmann, 2017), whereas glycolysis preferentially arises in the PV area (Braeuning et al., 2006; Jungermann and Katz, 1982). The above results indicate that PPARα and FXR may function in the liver through zonal activation.

The blood oxygen concentration exhibits a gradient decrease from PP area to PV area (Kietzmann, 2017), thus hepatocytes located in different parts of the liver cell plate are exposed to different microenvironments. Sox9^+^ hepatocytes constitutively reside in portal triads of healthy liver and are activated only in response to liver damage, and clonally labeled Sox9^+^ hepatocytes produce new hepatocytes and differentiate into bile duct cells (Font-Burgada et al., 2015). However, spatial and temporal coordination to Sox9^+^ hepatocytes by PP niches have not been well studied.

New bile duct cells could be supplied by preexisting bile duct cells or Sox9^+^ hepatocytes since Sox9-Cre^ERT2^ labels both Sox9^+^ hepatocytes and bile duct cells. But, few CK19^+^/BrdU^+^ cell was observed in the chronic CCl_4_ model of Sox9-Cre^ERT2^; Rosa26-mTmG mice suggesting new bile duct cells might be mainly supplied by Sox9^+^ hepatocytes. Our study indicated that Sox9^+^ hepatocytes constitutively reside in PP of healthy liver, which is related to its niches. The positive regulation of Sox9^+^ hepatocytes by PPARα activation and FAO promoted these hepatocytes exist in PP area of liver injury repair. The negative regulation of Sox9^+^ hepatocytes by FXR activation and glycolysis limited Sox9^+^ hepatocytes to PP area of the liver. PP niche is the key to steady residence of Sox9^+^ hepatocytes in PP area of healthy liver. The above results indicated that PPARα and FXR coordinated homeostasis of Sox9^+^ hepatocytes of healthy liver. Our data showed that PPARα activation increased FAO, OXPHOS, and ATP levels, thus promoting proliferation and differentiation of Sox9^+^ hepatocytes along PP-PV axis; however, FXR activation increased glycolysis, decreased OXPHOS and ATP production, therefore preventing the proliferation of Sox9^+^ hepatocytes along PP-PV axis by promoting self-renewal of Sox9^+^ hepatocytes. These results might be attributed to the change in niches of Sox9^+^ hepatocytes, and these findings were in line with the dynamic zonal metabolism. Our data offered a more feasible explanation that Sox9^+^ hepatocytes were differentially regulated in the two different zones (PP and PV) of the liver.

Energy metabolism plays important role in regulating stem cell function and fate (Zhang et al., 2012a). Studies have indicated that the mitochondrial dynamics and energy metabolism play important roles in cell reprogramming (Folmes et al., 2011) and mitochondrial morphology affects its function (Choi et al., 2020). Well-developed elongated mitochondria produce more energy or ATP than the immature globular mitochondria (Seo et al., 2020). Several studies have also suggested elongated mitochondria mainly rely on OXPHOS to produce energy (Rossignol et al., 2004), and globular mitochondria mainly produce energy through glycolysis (Collins et al., 2002). Another study reported that ATP produced by mitochondria affected stem cell pluripotency and promoted the expression and function of Sox9 in the cultured spinal cord astrocytes (Zhang et al., 2018). Consistent with above studies, our results revealed that PPARα activation promoted the FAO with mitochondria elongated, and that FXR activation enhanced the glycolysis with mitochondria becoming globular-shaped. Previous studies have found that OXPHOS is taking on increased importance in stem cell differentiation and proliferation (Chen et al., 2008). Our data showed that PPARα activation increased FAO, OXPHOS, and ATP levels, thus promoting proliferation and differentiation of Sox9^+^ hepatocytes. Consistent with the findings that glycolysis was key regulators of stem cell self-renewal (Arthur et al., 2019; Ryall et al., 2015), our results showed that FXR activation increased glycolysis, decreased OXPHOS and ATP production, therefore promoting self-renewal of Sox9^+^ hepatocytes.

It is now increasingly recognized that metabolic pathways influence epigenetic changes associated with proliferation, differentiation, and self-renewal (Ryall et al., 2015). Not only does metabolism provide ATP to maintain homeostasis and cell replication and intermediates that are essential for cell proliferation (Folmes et al., 2012), metabolism can directly influence cellular regulation and the epigenome (Chisolm and Weinmann, 2018; Ghosh-Choudhary et al., 2020). Epigenetic regulation plays important roles in the modulation of cell differentiation (Chisolm and Weinmann, 2018). Metabolism drives cell fate transition through the epigenetic regulation of gene transcription, a phenomenon called metaboloepigenetics (Harvey and Rathjen, 2016). It would be better to invest whether Sox9^+^ hepatocytes fate determination is dependent on metaboloepigenetics or not in the future.

Overall, PPARα activation increased the expression of Sox9, but FXR activation inhibited the expression of Sox9, indicating intersecting and complementary genomic circuits in which PPARα and FXR regulated the expression of Sox9. The Sox9^+^ hepatocytes niches exhibited strong adaptability to maintain a dynamic balance after chronic liver injury. In this process, PPARα activation promoted proliferation and differentiation of Sox9^+^ hepatocytes, whereas FXR activation prevented the proliferation of Sox9^+^ hepatocytes by promoting self-renewal or quiescent state of Sox9^+^ hepatocytes, which might be the mechanisms underlying the proper termination of liver regeneration. The opposite regulations of Sox9^+^ hepatocytes by PPARα and FXR prevented the excessive proliferation of Sox9^+^ hepatocytes, which explain why propagation of Sox9^+^ hepatocytes could restore tissue function and simultaneously avoid tumorigenesis caused by excessive proliferation despite their high regeneration potential.

Our research identified PPARα and FXR as potential therapeutic targets for liver regeneration. This study provides useful information for maintaining homeostasis of liver or other metabolic tissues. Our findings will be of great significance for exploring pathogenesis mechanism of multiple human diseases, as well as therapeutic strategies.

### Limitations of the study

Cells constantly adjust their metabolic state in response to various changes in extracellular environment, and the intermediary metabolites can influence the regulation of the epigenome, including histone methylation and acetylation (Ryall et al., 2015; Tsogtbaatar et al., 2020). Histone methylation plays a fundamental role in regulating the fate of embryonic stem cells (Vougiouklakis et al., 2017). Acetylation is dependent on acetyl-CoA produced by glycolysis, which is critical for the regulation of cell fate (Moussaieff et al., 2015). This cross talk between metabolism and epigenetics contribute to stem cell fate determination (Tsogtbaatar et al., 2020). We provide evidence that energy metabolism to regulate Sox9^+^ hepatocyte fate. However, the relationship among the metabolism, epigenetics, and cell differentiation may be experimentally validated in Sox9^+^ hepatocytes fate determination in future studies.

## STAR★Methods

### Key resources table

**Table:** 

REAGENT or RESOURCE	SOURCE	IDENTIFIER
**Antibodies**		

Mouse anti-FXR	Santa Cruz	Cat#sc-25309;RRID:AB_628039
Mouse anti-PPARα	Santa Cruz	Cat#sc-398394;RRID:AB_2885073
Mouse anti-BrdU	Servicebio	Cat#GB12051
Mouse anti-Notch1	Santa Cruz	Cat#sc-376403;RRID:AB_11149738
Rabbit anti-Sox9	Millipore	Cat#AB5535;RRID:AB_2239761
Mouse anti-Hnf4α	Abcam	Cat#ab41898;RRID:AB_732976
Rabbit anti-GFP	Proteintech	Cat#50430-2-AP
Mouse anti-Ck19	Servicebio	Cat#GB12197
Rabbit anti-CK19	Abcam	Cat#ab52625;RRID:AB_2281020
Goat anti-Rabbit IgG (H+L) Cross-Adsorbed Secondary Antibody,Alexa Fluor 555	Invitrogen	Cat#A-21428;RRID:AB_2535849
Goat anti-Mouse IgG (H+L) Cross-Adsorbed Secondary Antibody,Alexa Fluor 555	Invitrogen	Cat#A-21422;RRID:AB_2535844
Goat anti-Mouse IgG (H+L) Cross-Adsorbed Secondary Antibody,Alexa Fluor 488	Invitrogen	Cat#A11001;RRID:AB_2534069
Mouse anti-GAPDH	Proteintech	Cat#60004-I-Ig
Biotin-conjugated Affinipure Goat Anti-Rabbit IgG(H+L)	Proteintech	Cat#SA00004-2
Biotin-conjugated Affinipure Goat Anti-Mouse IgG(H+L)	Proteintech	Cat#SA00004-1

**Chemicals, peptides, and recombinant proteins**		

GW7647	Cayman	Cat#10008613
GW4064	MCE	Cat#HY-50108
tamoxifen	Sigma	Cat#T5648
DAPI	Abcam	Cat#104139
BrdU	Sigma	Cat#B5002

**Critical commercial assays**		

Luminescent ATP Detection Assay Kit	Beyotime	Cat#S0027
Glycolysis Cell-based Assay Kit	Cayman	Cat#600450
Oxygen Consumption Rate Assay Kit 600800	Cayman	Cat#600800
electrophoretic mobility shift assay (EMSA) Kit	Beyotime	Cat#GS009
ChIP Assay kit	Beyotime	Cat#P2078
pGL3-basic vector	Promega	Cat#E1751
phRL-TK	Promega	Cat#E2241
Lipofectamine 2000	Invitrogen	Cat#11668019
dual-luciferase assay kit	Promega	Cat#E1910
GPT/ALT kit	Nanjing jiancheng	Cat#C009-2
GOT/AST kit	Nanjing jiancheng	Cat#C010-2
Hematoxylin and Eosin Staining Kit	Beyotime	Cat#C0105S
RNAscope Multiplex Fluorescent Reagent Kit v.2	Advanced Cell Diagnostics	Cat#323110
RNAscope® H_2_0_2_ and Protease Reagents	Advanced Cell Diagnostics	Cat#322381

**Experimental models: Cell lines**		

HepG2	ATCC	Cat#HB-8065
Hep1-6	ATCC	Cat#CRL-1830
primary mouse hepatocytes	This paper	N/A

**Experimental models: Organisms/strains**		

FXR−/− mice	Jackson Laboratory	strain name: B6.129X1(FVB)-Nr1h4tm1Gonz/J, stock number 007214
PPARα−/− mice	Jackson Laboratory	strain name: B6;129S4-Pparαtm1Gonz/J, stock number 008154
Sox9-CreERT2 mice	Jackson Laboratory	strain name: STOCK Tg (Sox9-cre/ERT2) 1Msan/J, stock number 018829
Rosa26-mTmG mice	Jackson Laboratory	B6.129(Cg)-Gt (ROSA)26Sortm4(ACTB-tdTomato,-EGFP)Luo/J, stock number 007676
C57BL/6 mice	Laboratory Animal Center	N/A

**Oligonucleotides**		

See Tables S1 and S2		

**Recombinant DNA**		

pGL3-basic-Sox9-promoter vector	This paper	N/A
pGL3-basic-Sox9- mutation vector	This paper	N/A

**Software and algorithms**		

Image Pro Plus v.7	Image Pro Plus	https://image-pro-plus.software.informer.com/
GraphPad Prism 6	GraphPad Prism	https://www.graphpad.com/
Zen 2.3 lite	Carl Zeiss Microscopy	https://www.zeiss.com/microscopy/int/products/microscope-software.html

### Resource availability

#### Lead contact

Further information and requests for resources and reagents should be directed to and will be fulfilled by the lead contact, Lisheng Zhang (lishengzhang@mail.hzau.edu.cn).

#### Materials availability

This study did not generate new unique materials.

### Experimental model and subject details

#### Animal studies

The animals in this study were against a C57BL6/J background. The strains and animals used in our experiment were purchased from the Jackson Laboratory and bred in house, including FXR^−/−^ mice (strain name: B6.129X1(FVB)-Nr1h4^tm1Gonz^/J, stock number 007214), PPARα^−/−^ mice (strain name: B6;129S4-Pparα^tm1Gonz^/J, stock number 008154), Sox9-Cre^ERT2^ mice (strain name: STOCK Tg (Sox9-cre/ERT2) 1Msan/J, stock number 018829) and Rosa26-mTmG mice (B6.129(Cg)-Gt (ROSA)26Sor^tm4(ACTB-tdTomato,-EGFP)Luo/^J, stock number 007676). The wild-type (WT) mice (male, 6 to 8-week-old, n=5 per group), PPARα^−/−^ mice (male, 6 to 8-week-old, n=5 per group), and FXR^−/−^ mice (male, 6 to 8-week-old, n=5 per group) were orally treated with Vehicle (4:1 of PEG-400 and Tween 80), GW7647 (Cayman,10008613, 5mg/kg body weight) or GW4064(MCE, HY-50108 50mg/kg body weight) twice a day for two days. In CCl_4_-induced injury experiments, CCl_4_ was diluted with paraffin oil (Aladdin, C116023) to obtain 20% final concentration, and the diluted CCl_4_ was injected into mice at the dose of 2ml/kg body weight. The WT mice (male, 6 to 8-week-old, n=5 per group), PPARα^−/−^ mice (male, 6 to 8-week-old, n=5 per group), or FXR^−/−^ mice (male, 6 to 8-week-old, n=5 per group) were received intraperitoneal paraffin oil injection (control group) or CCl_4_ injection (at 1:4 of CCl_4_ to paraffin oil, 2ml/kg body weight) twice per week for four weeks and these mice were orally gavaged with either Vehicle (4:1 of PEG-400 to Tween 80), GW7647 (5mg/kg body weight), or GW4064 (50mg/kg body weight) four times a week for four weeks. In lineage tracing experiments, Sox9-Cre^ERT2^ mice were crossed with Rosa26-mTmG mice to obtain Sox9-Cre^ERT2^; Rosa26-mTmG heterozygous mice. These heterozygous mice were intraperitoneally injected with a single dose of tamoxifen (Sigma, T5648, 100mg/kg body weight/day) once per day for three days before treatment. The Sox9-Cre^ERT2^; Rosa26-mTmG mice (male, 6 to 8-week-old, n=5 per group) were received intraperitoneal paraffin oil injection (control group) or CCl_4_ injection (at 1:4 of CCl_4_ to paraffin oil, 2ml/kg body weight) twice per week for four weeks and these mice were orally gavaged with either Vehicle (4:1 of PEG-400 to Tween 80), GW7647 (5mg/kg body weight), or GW4064 (50mg/kg body weight) four times a week for four weeks. Bromodeoxyuridine (BrdU) (Sigma, B5002) was injected at the dose of 50mg/kg body weight twice per day for two days before sacrifice. After mice were killed, liver tissues were frozen in liquid nitrogen or fixed in 10% buffered formalin and embedded in paraffin or optimal cutting temperature (OCT) compound and then serum was collected. All procedures followed the Huazhong Agricultural University Guidelines for the Care and Use of Laboratory Animals.

### Method details

#### Isolation of primary mouse hepatocytes and cell sorting

The WT mice (male, 6 to 8-week-old) were anesthetized with Avertin (Sigma, T48402, 240mg/kg body weight) by intraperitoneal injection. The liver perfusion was done by injecting needle into the portal vein and providing the following solutions sequentially: 50 mL of EBSS (Sigma, E6276) supplemented with 0.5mM EGTA (Sigma, E3889) and then 50 mL of HBSS (Sigma, H1641) supplemented with 100 U/mL of Collagenase IV (Invitrogen, 17101–015), and 0.05 mg/mL of Trypsin inhibitor (Sigma, T2011). The perfused liver was carefully taken out, put onto a petri dish, added 25 mL of hepatocyte wash media (Invitrogen,17704–024), and massaged with two cell scrapers until the liver has become apart with only connective tissue left behind. Dissociated cells were passed through funnel with mesh into 50 mL of centrifugal tube. After centrifugation at 900 rpm for 5 min, cell pellet was resuspended in hepatocyte wash media, which were carefully overlaid percoll (Sigma, P4937) solution (50%). After centrifugation at 900 rpm for 10 min, harvested cell pellet was washed twice with hepatocyte wash media, and then suspended in Williams’ E medium (Invitrogen, 12551–032) supplemented with 10% FBS, 1% penicillin/streptomycin and were seeded into 6-well, 24-well, 96-well plates or Laser confocal dish.

For cell sorting, liver single cells were isolated from Sox9-Cre^ERT2^; Rosa26-mTmG mice (male, 6 to 8-week-old) by collagenase digestion perfusion technique. Cell suspensions were measured on a BC- FC500 for cell sorting (Sinha and Lowell, 2016).

#### Cell culture

The cell lines used in this study included HepG2 cells (ATCC HB-8065), Hep1-6 cells (ATCC CRL-1830), primary mouse hepatocytes and GFP^+^ primary mouse hepatocytes. Cells were seeded into 6-well, 24-well plates, 96-well plates or Laser confocal dish, and grown in high glucose DMEM (Hyclone, Logan, UT, USA) supplied with 10% (vol/vol) fetal bovine serum (FBS; Gibco BRL, Grand Island, NY, USA) and 1% (vol/vol) penicillin-streptomycin. Cells were cultured in a carbon dioxide incubator (Thermo Scientific, USA) with 5% CO_2_ at 37°C. HepG2 cell, Hep1-6 cell or primary mouse hepatocytes respectively treated with DMSO (Veh), GW7647(5μM), or GW4064 (10μM) for 24h. GFP^+^ primary mouse hepatocytes incubated with BrdU (10μM) for 48h, meanwhile, treated with DMSO (Veh), GW7647 (5μM), or GW4064 (10μM) for 48h to immunofluorescent analysis. GFP^+^ primary mouse hepatocytes treated with DMSO (Veh), GW7647 (5μM), or GW4064 (10μM) for 24h to immunofluorescent analysis. GFP^+^ primary mouse hepatocytes treated with DMSO (Veh), GW7647 (5μM), or GW4064 (10μM) for 24h to transmission electron microscopic. Primary mouse hepatocytes treated with DMSO (Veh), GW7647 (5μM), or GW4064 (10μM) for 24h to ATP concentration, O_2_ consumption and Glycolysis measurements.

#### Electron microscopy

GFP^+^ primary mouse hepatocytes treated with DMSO (Veh), GW7647 (5μM), or GW4064 (10μM) for 24h to transmission electron microscopic. For transmission electron microscopic (TEM) observations, the samples were fixed in 4% paraformaldehyde and 2.5% glutaraldehyde in 0.1M phosphate buffer for overnight. After washing in 0.1M phosphate buffer, the samples were postfixed with 1% osmium tetroxide in same buffer for 1h. Then the samples were dehydrated with a series of the graded ethyl alcohol. The samples were embedded in Epon 812 and then polymerization was performed at 60°Cfor 3 days. Ultrathin sections (60∼70nm) were obtained by ultramicrotome (Leica UC7, Germany). Images were acquired with transmission electron microscope (FEI, TECNAI G2 20 TWIN, USA) after double staining with uranium acetate and lead citrate.

#### ATP measurement, glycolysis and O_2_ consumption measurement

Luminescent ATP Detection Assay Kit (Beyotime, S0027) is used to measure the level of ATP within the cell or liver tissue. Cell or liver tissue were lysed, added the firefly luciferase, and luciferase activity was measured using Fluoroskan Ascent FL (Thermo Scientific, USA). Primary mouse hepatocytes treated with DMSO (Veh), GW7647 (5μM), or GW4064 (10μM) for 24h to Glycolysis measurements. Glycolysis Cell-based Assay Kit (Cayman, 600450) is used to measure the glycolysis within primary mouse hepatocytes. Cayman's Glycolysis Cell-Based Assay Kit provides a colorimetric method for detecting L-lactate, the end product of glycolysis, produced and secreted by cultured cells. Glycolysis measurement on primary mouse hepatocytes were measured using Fluoroskan Ascent FL (Thermo Scientific, USA). Primary mouse hepatocytes treated with DMSO (Veh), GW7647 (5μM), or GW4064 (10μM) for 24h to O_2_ consumption measurements. Oxygen Consumption Rate Assay Kit 600800 (Cayman, 600800) is used to measure the O_2_ consumption within primary mouse hepatocytes. Cayman's cell-based Oxygen Consumption Rate Assay Kit utilizes this newly developed phosphorescent oxygen probe to measure oxygen consumption rate in living cells. O_2_ consumption measurement on primary mouse hepatocytes were measured using Fluoroskan Ascent FL (Thermo Scientific, USA).

#### RNA isolation and QRT-PCR

The RNAiso Plus (Takara, 9109) was used to isolate total RNA. Then the first-strand cDNA was synthesized using the PrimeScript RT Reagent Kit with gDNA Eraser (Takara, RR047A). Real-time PCR was performed using the MonAmp™ SYBR® Green qPCR Mix (MQ10201S, Low ROX). Real-time PCR was measured using QuantStudio™ 3 Real-Time PCR Instrument (Applied Biosystems, USA). The relative levels were calculated using the comparative-Ct method (2^-ΔΔCt^ method). The primer sequences are listed in Table S1.

#### EMSA

Nuclear extracts were prepared from GW4064 or GW7647-treated livers using the Active Motif Nuclear Extract Kit (Active Motif, 40010 & 40410). The protein concentrations of nuclear extracts were determined using the BCA protein assay kit (Beyotime, P0009). Two single-stranded oligo nucleotides (Sangon, China) corresponding to the promoter were synthesized and annealed into double strands. The DNA binding activity of PPARα or FXR was detected by a chemiluminescent electrophoretic mobility shift assay (EMSA) Kit (Beyotime GS009). The sequences are listed in Table S1.

#### ChIP

ChIP assays were performed using the ChIP Assay kit (Beyotime, P2078). Hep1-6 cells or mice were treated with GW7647 or GW4064. Subsequently, the treated Hep1-6 cells or liver tissues were sonicated and then immunoprecipitated with the antibody against FXR (1:100 dilution Santa Cruz, sc-25309) or PPARα (1:100 dilution Santa Cruz, sc-398394) with IgG (1:100 dilution Santa Cruz, sc-2027) as a negative control. The captured chromatin was eluted and un-crosslinked, and the DNA was recovered. The ChIP-isolated DNA was subjected to PCR amplification using the primer pair spanning the Sox9 promoter region. The primer sequences are listed in Table S1.

#### Molecular cloning and cell-based luciferase reporter assay

Putative PPREs and FXRE in the Sox9 promoter region was predicted using an online algorithm (NUBIScan: http://www.nubiscan.unibas.ch/). Based on this prediction, the Sox9 promoter fragments were amplified by PCR using mouse genomic DNA as a template. Afterward, the amplified fragments were separately inserted into the pGL3-basic vector (Promega, E1751). The recombinant plasmid was performed site-directed mutation of the elements. The mouse PPARα, mouse FXR and mouse RXR expression vectors were created in our lab. In luciferase reporter assays, the above plasmids together with the Renilla luciferase expression vector phRL-TK (Promega, E2241) were individually co-transfected into HepG2 or Hep1-6 cells, using Lipofectamine 2000 (Invitrogen, 11668019). After 6h incubation, the cells were treated with DMSO (Veh), GW7647(1μM), or GW4064 (1μM) for 24h. The cells were then collected for luciferase activity detection by using the dual-luciferase assay kit (Promega, E1910). The enzymatic activity of luciferase was measured by using a Fluoroskan Ascent FL (Thermo Scientific, USA). Firefly luciferase activity was normalized with renilla luciferase activity as internal control. The primer sequences are listed in Table S1.

#### Serum transaminase levels and histological analysis

Plasma was collected from blood after centrifugation (at 3,000 rpm) for 10 min at 4°C. Plasma ALT, AST were determined to evaluate liver injury using a Multiskan MK3 microplate reader (Thermo Electron Corporation, USA) and GPT/ALT kit (Nanjing jiancheng, C009-2) or GOT/AST kit (Nanjing jiancheng, C010-2). Liver tissues were immobilized with 4% PFA, dehydrated, embedded in paraffin, sectioned at 5 μm, and processed for H&E (Beyotime, C0105S).

#### Immunofluorescent analysis

For immunofluorescent staining, cells were fixed, permeabilized, and processed for immunostaining with primary antibody BrdU (1:100 Servicebio, GB12051), Notch1 (1:100 dilution Santa Cruz, sc-376403). Incubate at 4°C for 12h. Alexa Fluor 555(Invitrogen, A-21422) was used as secondary antibodies. Incubate at room temperature for 2h. DAPI (Abcam, ab104139) counterstaining was employed to demonstrate nuclei. Images were acquired with a laser scanning confocal microscope (LSM710, Carl Zeiss Microscopy), and analyzed by Zen software (Zen 2.3 lite, Carl Zeiss Microscopy) with fixed parameters. For double immunohistochemical staining, paraffin-embedded liver sections were used. Samples were fixed and permeabilized, saturated, and processed for immunostaining with primary antibody Sox9(1:100 dilution Millipore, AB5535)/Hnf4α(1:100 dilution Abcam, ab41898), Sox9(1:100 dilution)/BrdU(1:100 dilution), GFP(1:200 dilution Proteintech, 50430-2-AP)/Hnf4α(1:100 dilution), GFP(1:200 dilution)/BrdU(1:200 dilution), GFP(1:200 dilution)/Ck19(1:200 dilution Servicebio, GB12197), Ck19(1:200 dilution Abcam ab52625)/BrdU(1:200 dilution), GFP(1:200 dilution)/Notch1(1:100 dilution Santa Cruz, sc-376403). Incubate at 4°C for 12h. Alexa Fluor 555(1:500 dilution Invitrogen, A-21428) and Alexa Fluor 488(1:500 dilution Invitrogen, A11001) were used as secondary antibodies. Incubate at room temperature for 2h. DAPI (Abcam, ab104139) counterstaining was employed to demonstrate nuclei. Images were acquired with a laser scanning confocal microscope (LSM710, Carl Zeiss Microscopy), and analyzed by Zen software (Zen 2.3 lite, Carl Zeiss Microscopy) with fixed parameters.

#### Western blots

For whole-cell protein extraction, liver tissues were prepared in lysis buffer (Beyotime, P0013B). Protein lysates were separated by SDS-PAGE. Next, the gel was transferred to polyvinylidene difluoride membranes (Millipore, IPVH00010). After being blocked with 5% skimmed milk, the membranes were incubated overnight with the anti-Sox9 (1:200 dilution Millipore,AB5535), anti-GAPDH (1:500 dilution Proteintech, 60004-I-Ig) at 4°Cfor 12h. Then, the membranes were incubated with the corresponding secondary antibodies (1:1000 dilution Proteintech SA00004-2; 1:1000 dilution Proteintech SA00004-1;) at room temperature for 1.5h. Finally, the membranes were visualized with enhanced chemiluminescence (Bio-Rad, USA).

#### *In situ* hybridization (ISH)

*In situ* detection of Sox9 and Pck1 RNA transcripts was carried out on OCT-embedded tissue sections using the RNAscope Multiplex Fluorescent Reagent Kit v.2 (Advanced Cell Diagnostics, 323110). Prepared cryosections were fixed in formaldehyde for a quarter of an hour at 4°C, dehydrated, and pre-treated in hydrogen peroxide for 10 min, followed by half an hour digestion in protease III (Advanced Cell Diagnostics, 322381). Subsequently, signal was pre-amplified and amplified in terms of the directions. The resultant sections were counterstained using mounting medium with DAPI. Acquisition of ISH signal by laser confocal microscope (LSM710, Carl Zeiss Microscopy), and analyzed by Zen software (Zen 2.3 lite, Carl Zeiss Microscopy) with fixed parameters. The probe information for RNAscope assay is listed in Table S2.

#### Software–intensity measurement

Image Pro Plus (Image Pro Plus v.7: Media Cybernetics; Bethesda, MD), as an analysis program, was used to analyze and quantify data from photomicrographs. In this study, the analyses were performed as follows: Integrated Optical Density (IOD) Image Pro Plus was used to quantify the intensity of probes binding to the structures. We used the confocal series to calculate the total binding intensity of the probes (IOD-intensity value). At least three confocal images were used for each experiment treatments and their average were plotted and analyzed (Karamichos et al., 2011).

### Quantitative and statistical analysis

#### Statistical analysis

Statistical analyses were performed using the GraphPad Prism 6 (GraphPad). Data are expressed as means ± SD. Comparisons between two groups were performed using the two-tailed Student’s t-test. Comparisons between multiple groups were performed using ordinary one-way ANOVA with the Dunnett's multiple comparison test. Statistical significance was presented at the level of ∗p < 0.05, ∗∗p < 0.01, ∗∗∗p < 0.001.

## Data Availability

All data produced or analyzed for this study are included in this published article and its supplemental information files. This paper did not report original code. Any additional information required to reanalyze the data reported in this paper is available from the lead contact upon request.
