# Supplementary material for: Metabolic nuclear receptors coordinate energy metabolism to regulate Sox9+ hepatocyte fate
Source: iScience. 2021 Aug 19;24(9):103003. doi: 10.1016/j.isci.2021.103003 (PMC8417399; doi:10.1016/j.isci.2021.103003)
Supplement: Document S1. Figures S1–S6 and Tables S1 and S2 [file mmc1.pdf]

iScience, Volume *24*

Supplemental information

Metabolic nuclear receptors coordinate energy metabolism to regulate Sox9$^+$ hepatocyte fate

Shenghui Liu, Dan Qin, Yi Yan, Jiayan Wu, Lihua Meng, Wendong Huang, Liqiang Wang, Xiangmei Chen, and Lisheng Zhang

Supplemental information

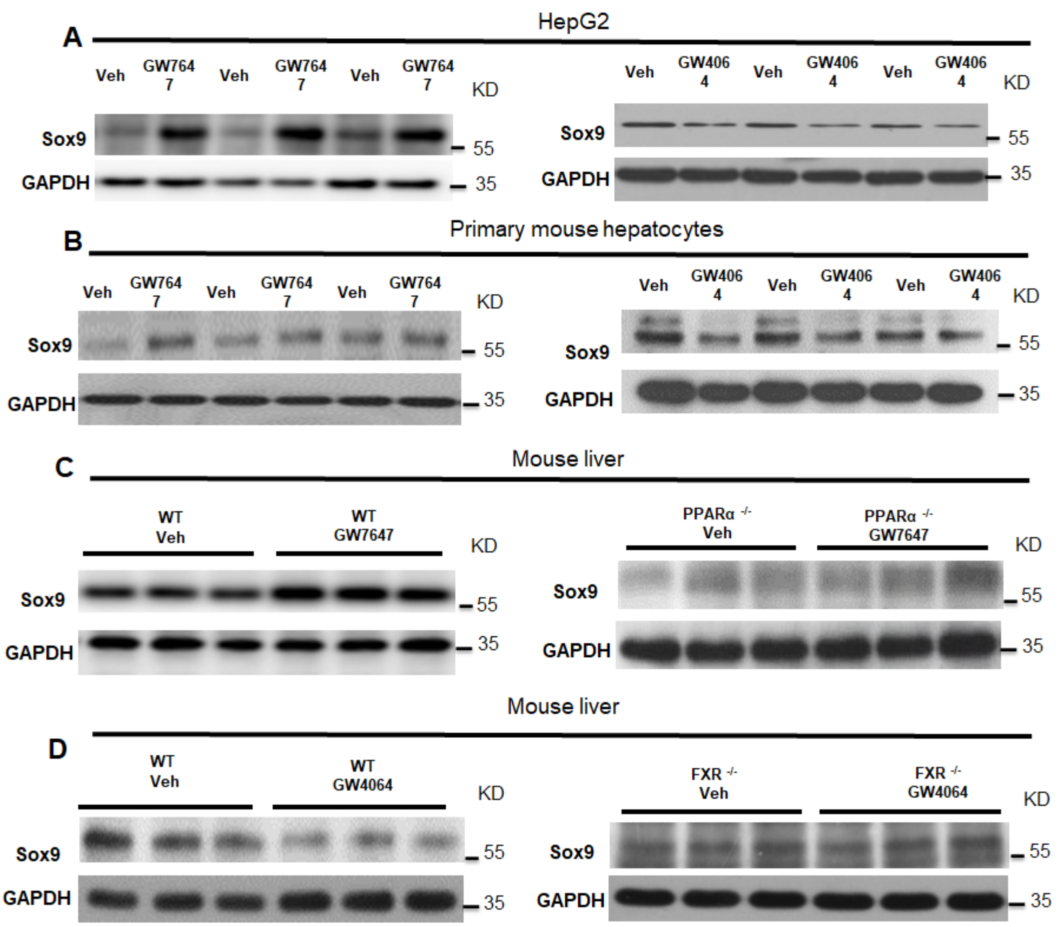

Figure S1. Activation of PPARα or FXR regulates the expression of Sox9 in vitro and in vivo, related to Figure 1 and 2.

(A) Sox9-Immunoblot analysis in HepG2 cells.

(B) Sox9-Immunoblot analysis in primary mouse hepatocytes cells.

(C) Sox9-Immunoblot analysis of liver samples.

(D) Sox9-Immunoblot analysis of liver samples.

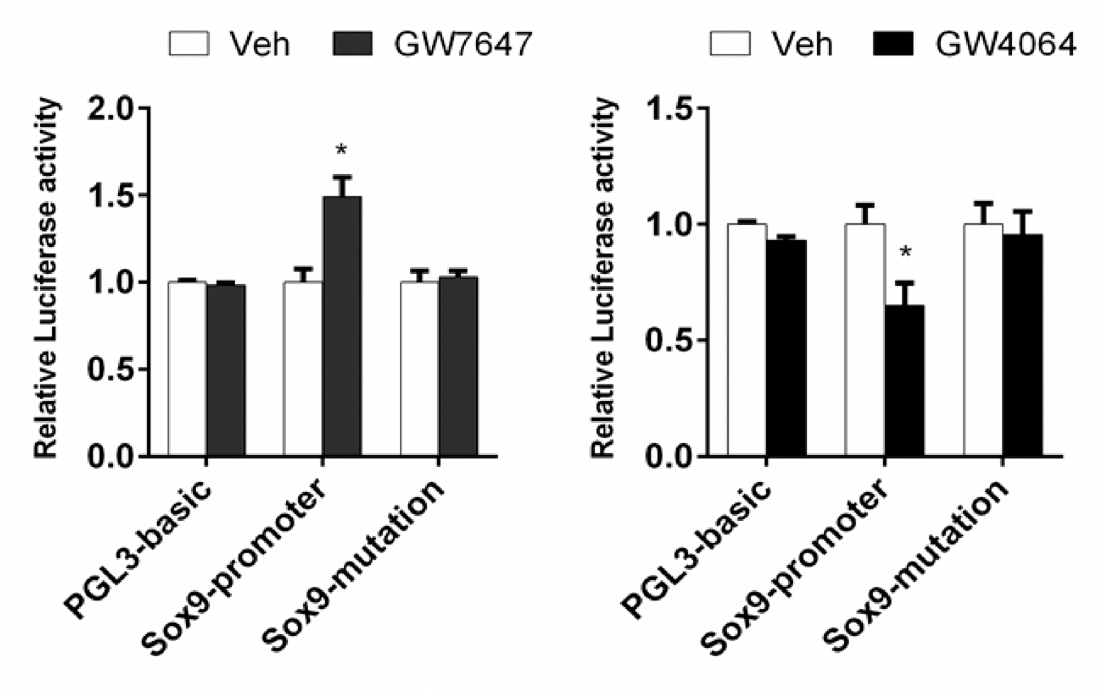

Figure S2. Sox9 is a target of PPARα and FXR, related to Figure 3.

Functional role of IR9 motif in the regulatory region of mouse Sox9 for PPARα and FXR activity in Hep1-6 cells. Data are expressed as means ± SD. Comparisons between two groups were performed using the two-tailed Student's t-test. Significant difference is presented at the levels of $^*p < 0.05$ and $^{**}p < 0.01$.

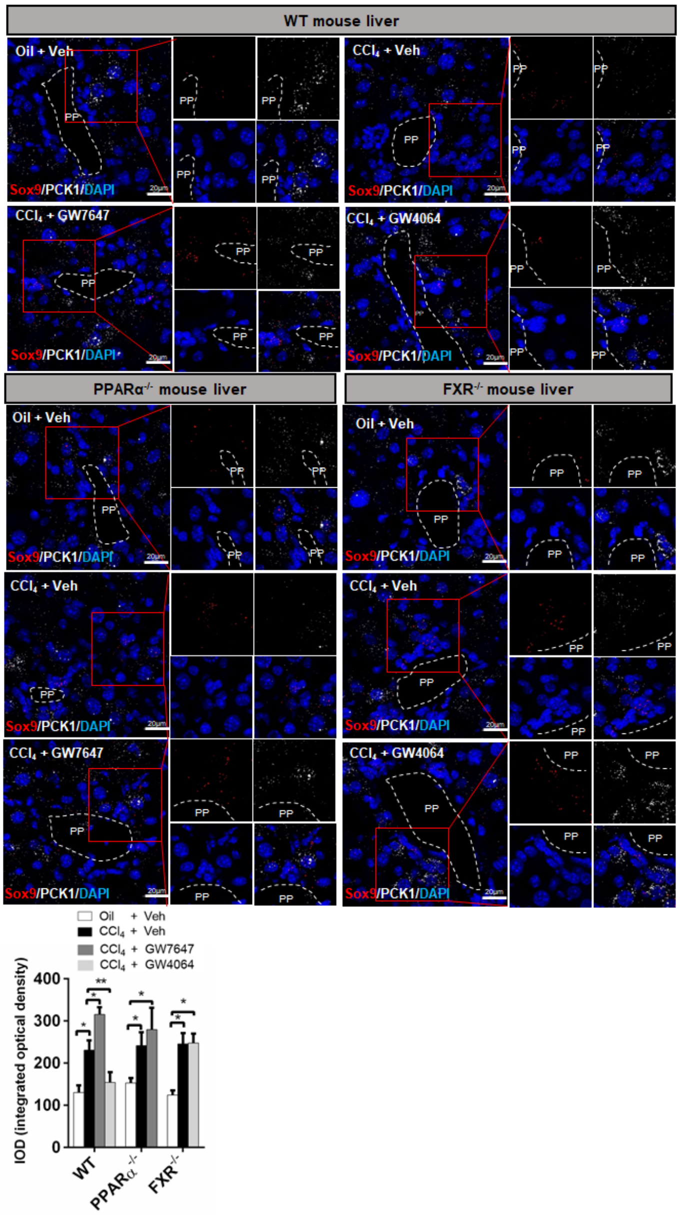

Figure S3. Activation of PPARα or FXR regulates the expression of Sox9 in vivo, related to Figure 4.

Representative images from RNAscope® assays for Sox9 mRNA levels in the indicated groups. Graph show quantification of the integrated optical density (IOD) in the indicated groups (n = 5). Scale bar represents 20μm. Data are expressed as means ± SD. Comparisons between multiple groups were performed using ordinary one-way ANOVA with the Dunnett's multiple comparison test. Significant difference is presented at the levels of $^*p < 0.05$ and $^{**}p < 0.01$.

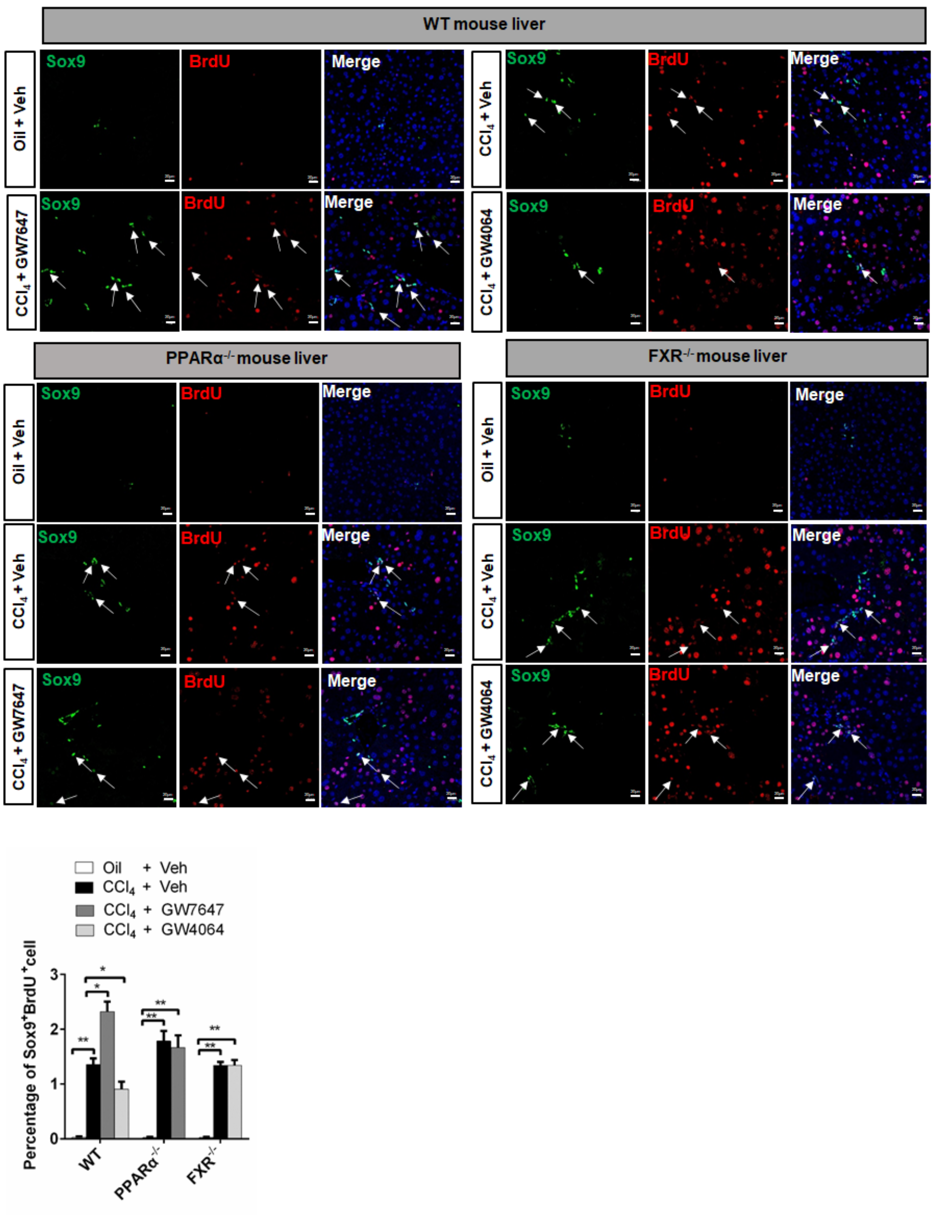

Figure S4. PPARα promotes, but FXR prevents proliferation of Sox9$^+$ BrdU$^+$ cells in CCl_4-induced chronic liver injury model, related to Figure 5. The model of CCl_4-induced chronic liver injury was described in Fig. 4*A*. Sox9/BrdU staining was performed. Arrows depict Sox9$^+$BrdU$^+$ cells. Graphs show percentages of Sox9$^+$BrdU$^+$ cells in the indicated groups (n=5). Scale bar represents 20μm. Data are expressed as means ± SD. Comparisons between multiple groups were performed using ordinary one-way ANOVA with the Dunnett's multiple comparison test. Significant difference is presented at the levels of $^*p < 0.05$ and $^{**}p < 0.01$.

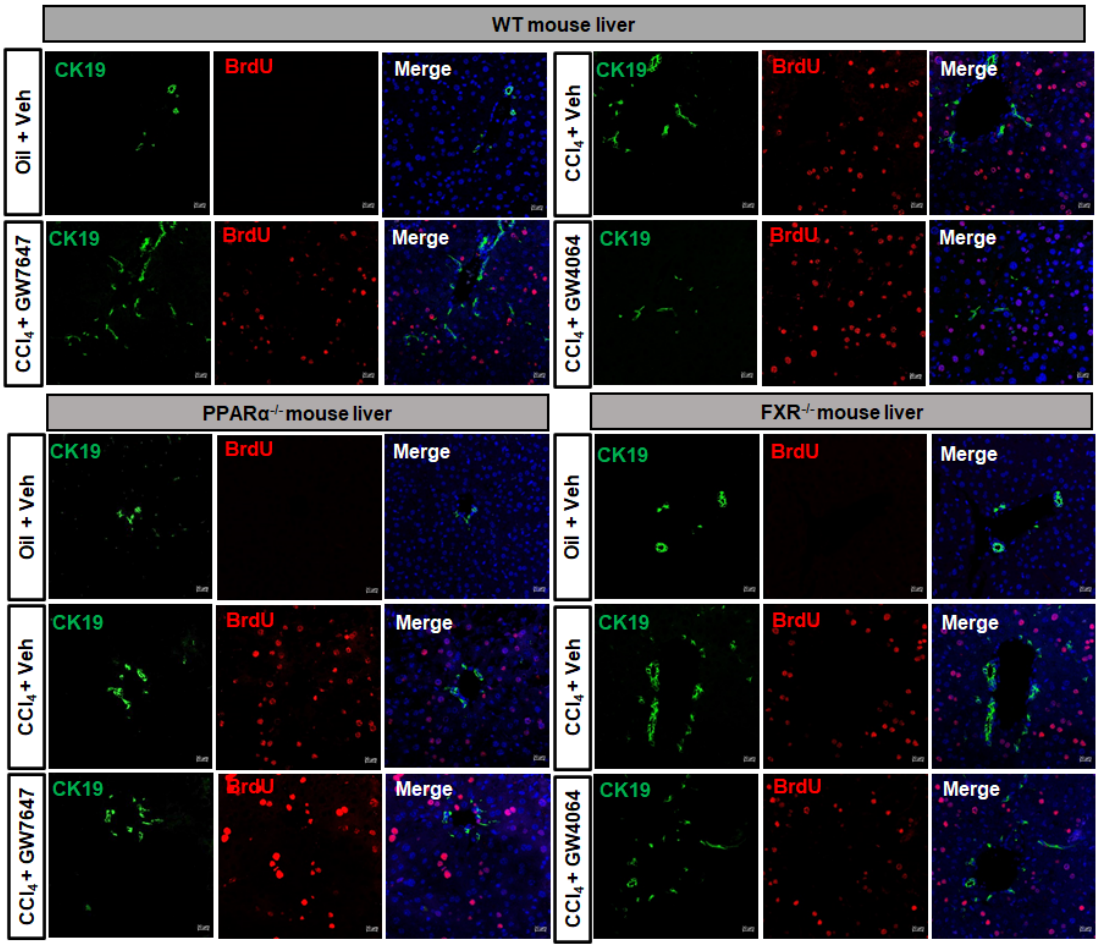

Figure S5. Few CK19⁺ BrdU⁺ cells in CCl_4-induced chronic liver injury model, related to Figure 5.

The model of CCl_4-induced chronic liver injury was described in Fig. 4*A*. CK19/BrdU staining was performed. Scale bar represents 20μm.

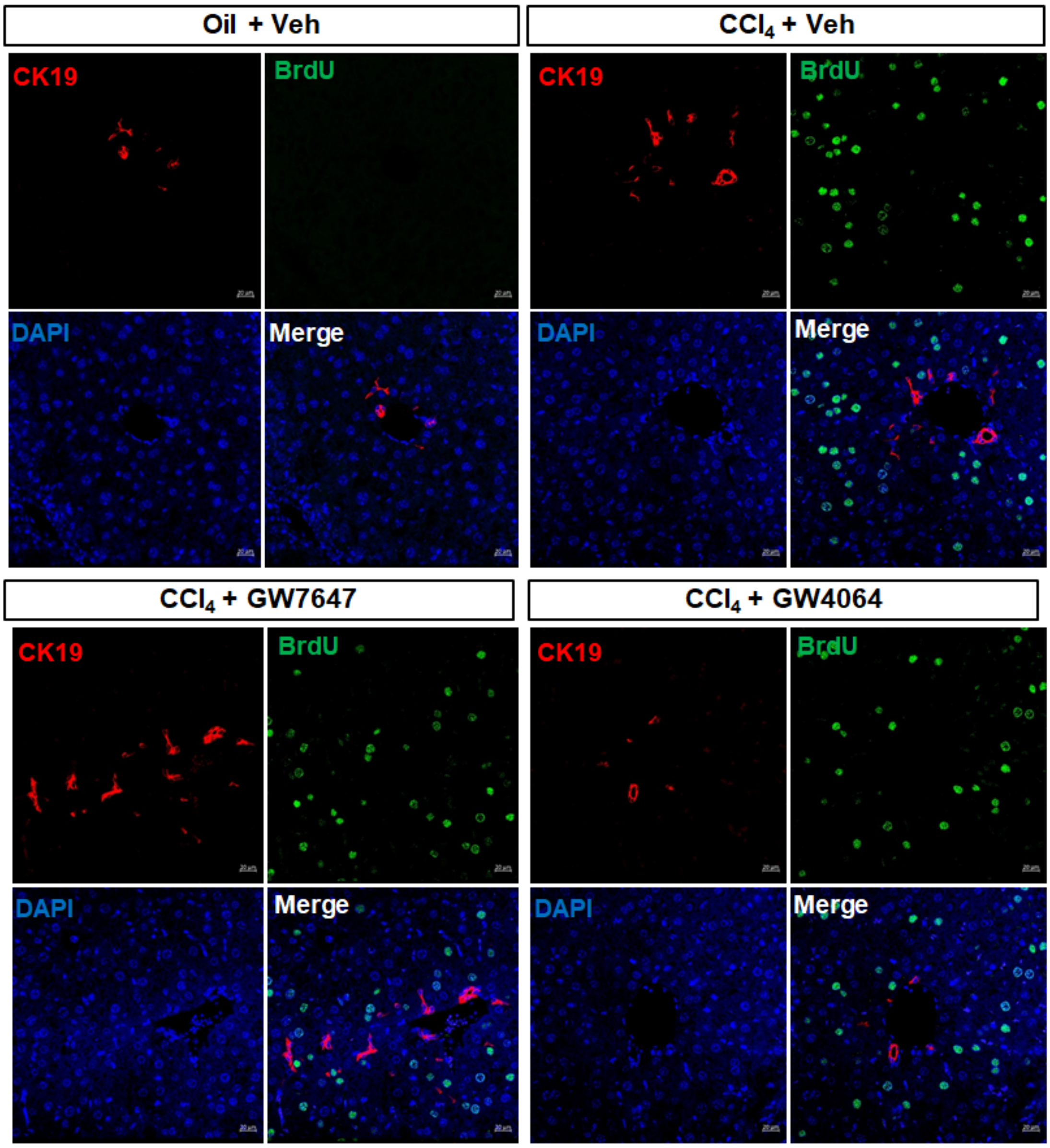

Figure S6. Few CK19⁺ BrdU⁺ cells after CCl4-induced chronic liver injury in Sox9-CreERT2; Rosa26-mTmG mice, related to Figure 7.

The model of CCl_4-induced chronic liver injury was described in Fig. 6B. CK19/BrdU staining was performed. Scale bar represents 20μm.

Table S1. The primer sequences for qPCR, EMSA, ChIP and Plasmid Construction, related to STAR Methods.

Name	Application	Sequence (5'-3')
H-Cpt1a-F	qPCR	CATTCAGGCAGCAAGAGC
H-Cpt1a-R	qPCR	CAGCAGCCGCCCATCATG
H-GAPDH-F	qPCR	CTCTGGTAAAGTGGATATTG
H-GAPDH-R	qPCR	GGTGGAATCATATTGGAAC
H- Acox1-F	qPCR	GTATGGAATCAGTCAGAACGC
H- Acox1-R	qPCR	CTTGTAAGATTCGTGGACCTC
H-SHP-F	qPCR	CCCAAGATGCTGTGACCTTT
H-SHP-R	qPCR	CCAGAAGGACTCCAGACAGC
H-Sox9-F	qPCR	ACCGACGAGCAGGAGAAGGG
H-Sox9-R	qPCR	GCGGATGCACACGGGGAACT
H-PDK4-F	qPCR	GTGATGTGGTAGCAGTGGTC
H-PDK4-R	qPCR	TCTATTGGTGTAAGGGAAGG
M-PDK4-F	qPCR	GAGCATCAAGAAAACCGT
M-PDK4-R	qPCR	ATAACCAAAACCAGCCAA
M-36B4-F	qPCR	TGGAGACAAGGTGGGAGCC
M-36B4-R	qPCR	CACAGACAATGCCAGGACGC
M-Acox1-F	qPCR	CTGAAATCAAGAGAAGCGAG
M-Acox1-R	qPCR	GAGAAAGTGGAAGGCATAGG
M-Cpt1a-F	qPCR	ACCTATTCGTCTTCTGGGAT
M-Cpt1a-R	qPCR	GTGTTGGATGGTGTCTGTCT
M-SHP-F	qPCR	CGATCCTCTTCAACCCAGATG
M-SHP-R	qPCR	AGGGCTCCAAGACTTCACACA
M-Sox9-F	qPCR	TGCAAGCTGGCAAAGTTGAT
M-Sox9-R	qPCR	TCAGTTCACCGATGTCCACG

Sox9-probe-F(Labeled)	EMSA	AGTTCAAGGTCGGCGTGGCCC
Sox9-probe-R(Labeled)	EMSA	GGGCCACGCCGACCTTGAACT
Sox9-probe-F(Mut)	EMSA	CTACCAAGGTCGGCGCATGCC
Sox9-probe-R(Mut)	EMSA	GGCATGCGCCGACCTTGGTAG
Sox9- probe-F(Cold)	EMSA	AGTTCAAGGTCGGCGTGGCCC
Sox9- probe-R(Cold)	EMSA	GGGCCACGCCGACCTTGAACT
M-Sox9-IR9-F	ChIP	AGTGGCGAGCCTGACGGTGTTTGTG
M-Sox9-IR9-R	ChIP	CCGTGGGCCACGCCGACCTTGAACT
M-SOX9-PGL3-F	Plasmid Construction	CGACGCGTCTCCCTACCTCAAGCCTG AAGAAT
M-SOX9- PGL3-R	Plasmid Construction	GAAGATCTGAACCAGCCGAGTCCTCC GACATG
M-SOX9-PGL3-Mut-F	Plasmid Construction	ACTTGTCCTACCAAGGTCGGCGCATG CCACGGGACCGGCCGCA
M-SOX9- PGL3-Mut -R	Plasmid Construction	TGCGGCCGGTCCCGTGGCATGCGCC GACCTTGGTAGGACAAGT

H: Human M: Mouse F: Forward R: Reverse

Table S2. The probe information for RNAscope® assay, related to STAR Methods.

Official symbol	Cat No.	SOURCE	Gene ID	Genbank nucleotide accession number	Channel	Detection Kit
Sox9	401051-C3	www.acdbio.com	20682	NM_011448.4	C3	RNAscope® Multiplex Fluorescent Reagent Kit v2
Pck1	458021	www.acdbio.com	18534	NM_011044.2	C1	
